# Recent Progress and Potential Biomedical Applications of Electrospun Nanofibers in Regeneration of Tissues and Organs

**DOI:** 10.3390/polym14081508

**Published:** 2022-04-07

**Authors:** AbdElAziz A. Nayl, Ahmed I. Abd-Elhamid, Nasser S. Awwad, Mohamed A. Abdelgawad, Jinglei Wu, Xiumei Mo, Sobhi M. Gomha, Ashraf A. Aly, Stefan Bräse

**Affiliations:** 1 Department of Chemistry, College of Science, Jouf University, P.O. Box 2014, Sakaka 72341, Al Jouf, Saudi Arabia; 2 Composites and Nanostructured Materials Research Department, Advanced Technology and New Materials Research Institute, City of Scientific Research and Technological Applications (SRTA-City), New Borg Al-Arab, Alexandria 21934, Egypt; ahm_ch_ibr@yahoo.com; 3 Research Center for Advanced Materials Science (RCAMS), King Khalid University, P.O. Box 9004, Abha 61413, Saudi Arabia; aawwad@kku.edu.sa; 4 Department of Pharmaceutical Chemistry, College of Pharmacy, Jouf University, Sakaka 72341, Al Jouf, Saudi Arabia; mhmdgwd@ju.edu.sa; 5 Key Laboratory of Science and Technology of Eco-Textile, Ministry of Education, College of Chemistry, Chemical Engineering and Biotechnology, Donghua University, Shanghai 201620, China; jw@dhu.edu.cn (J.W.); xmm@dhu.edu.cn (X.M.); 6 Chemistry Department, Faculty of Science, Cairo University, Giza 12613, Egypt; s.m.gomha@sci.cu.edu.eg; 7 Chemistry Department, Faculty of Science, Islamic University of Madinah, Madinah 42351, Saudi Arabia; 8 Chemistry Department, Faculty of Science, Organic Division, Minia University, El-Minia 61519, Egypt; ashrafaly63@yahoo.com; 9 Institute of Organic Chemistry, Organic Chemistry I, 76131 Karlsruhe, Germany; 10 Institute of Biological and Chemical Systems—Functional Molecular Systems (IBCS-FMS), 76344 Eggenstein-Leopoldshafen, Germany

**Keywords:** electrospinning, nanofibers, polymers, biomedical applications, regeneration of tissues

## Abstract

Electrospun techniques are promising and flexible technologies to fabricate ultrafine fiber/nanofiber materials from diverse materials with unique characteristics under optimum conditions. These fabricated fibers/nanofibers via electrospinning can be easily assembled into several shapes of three-dimensional (3D) structures and can be combined with other nanomaterials. Therefore, electrospun nanofibers, with their structural and functional advantages, have gained considerable attention from scientific communities as suitable candidates in biomedical fields, such as the regeneration of tissues and organs, where they can mimic the network structure of collagen fiber in its natural extracellular matrix(es). Due to these special features, electrospinning has been revolutionized as a successful technique to fabricate such nanomaterials from polymer media. Therefore, this review reports on recent progress in electrospun nanofibers and their applications in various biomedical fields, such as bone cell proliferation, nerve regeneration, and vascular tissue, and skin tissue, engineering. The functionalization of the fabricated electrospun nanofibers with different materials furnishes them with promising properties to enhance their employment in various fields of biomedical applications. Finally, we highlight the challenges and outlooks to improve and enhance the application of electrospun nanofibers in these applications.

## 1. Introduction

During the last century, the utilization of polymers has rapidly increased, leading to the development of techniques used to produce polymer fibers to satisfy the high-performance requirements of different industries and modern applications [1]. Nanofibrous materials are thin and long substances and they are 1D materials, with diameters ranging from 50.0 to 500.0 nm in length, having diameter ratios >1.0:200.0. These nanofibrous materials can be synthesized from polymer solutions or melts. Nanofiber structures have gained extensive attention and show large potential for various applications, due to their favorable and unique characteristics [2,3,4]. The unique properties of electrospun nanofiber materials, such as large surface area and porosity, in addition to adjustability of pore sizes, due to their similar morphology and extracellular matrix, gives nanofibers the edge, when compared with bulk materials, in the regeneration of tissues and organs. Nanofibers can resemble or mimic the human anatomy’s tissues and organs [4]. Therefore, nanofibers have been broadly utilized in various applications, such as in bone tissue engineering, nerve tissue repair or nerve regeneration, vascular tissue and skin tissue reconstruction. All these applications will be discussed hereafter. Due to the promising properties of nanofiber materials, they have attracted more and more attention. Therefore, various techniques were investigated and developed to synthesize and fabricate nanofibers, such as electrospinning, template synthesis, drawing, and self-assembly [4]. Among these techniques, electrospinning processes are the most well-known techniques used to fabricate a layer of nanofibers of diameters in the range of 3.0 nm–>5.0 mm with unique characteristics. The electrospinning technique employs electrostatic forces to produce nanofibers from a polymer solution, collected layer-by-layer to form a material. Both natural and synthetic polymers may be electrospun [5]. Integration of natural and synthetic polymers, the addition of ceramics, and investigation of core-shell fiber geometries all lead to control over various mechanical properties of the electrospun membrane, such as mechanical attributes, wettability, toxicity, etc. [6]. Additionally, polymer solution concentration, voltage, solvent, etc., can adjust the electrospinning parameters to control the fiber diameter and material porosity [7,8,9]. Meanwhile, membrane porosity increases with increase in the diameter of the nanofiber. Based on the unique features of the nanofiber, it is widely utilized in various applications, such as air filtration, water treatment, cosmetics, in the textile industry, and providing the active materials for photonics and electronics. Nanofiber features have also attracted a great deal of attention in biomedical fields, like wound dressing, tissue engineering, and regenerative medicine [1,10].

Tissue engineering is focused on fabricating scaffolds that can temporarily substitute for the native extracellular matrix (ECM), to guide and regenerate specific tissue functions. Tissue engineering technologies encompass rapidly advancing techniques, which combine features and advantages of biochemicals/biomaterials sciences and transplantation of cells to generate bioartificial tissues or organs to cure damage to skin [11], cartilage [12], bone [13], nerve [14], and vascular [15] tissues. Over the last three decades, much investigative work has been done to fabricate vascular grafts in small diameter for clinical applications, but most of the attempts failed or were not satisfactory for applications. Therefore, new cell-based approaches have been investigated to fabricate tissue-engineered scaffolds similar to native ECM [16]. Electrospinning technology provides several interesting characteristics to the design and manufacture of scaffolds, with topography in the micro- and nano-scales, as scaffolding biomaterials for tissue repair and regeneration [17]. Electrospun nanofiber scaffolds (ENFSs) also provide internal porous network structures similar to the natural extracellular matrix (ECM), which supports the growth of cells, tissues, and organs. To fulfill these features, ENFSs must be biocompatible, have a high surface area, high porosity, gas permeability, possess appropriate mechanical properties, and transfer bioactive molecules that support cell growth, spreading, proliferation, migration, infiltration, and attachment. These promising and unique characteristics of electrospun nanofibrous structures promote their stabilities and versatilities in surface functionality, leading to their being perceived as significant biomaterials. They have gained great attention in regards to bone cell proliferation, nerve regeneration, vascular tissue and skin tissue engineering applications [17,18,19]. Furthermore, a tissue engineering scaffold should be biodegradable, have low immunogenicity, be economical, easy to process, and commercially available. Moreover, nanofibers possess elasticity in the functionalization of their surfaces, where they can be modified by applying pretreatment or post-treatment strategies. In the pretreatment approach, the most widely utilized modifiers are directly blended with spinning materials, and the modifier will already be present in the nanofiber. On the other hand, in post-treatment, the nanofiber is first prepared and then treated with the modifying agent, where only the surface is functionalized [20].

Nanoparticles suffer from aggregations in liquid during use forming a slurry, and these powders are difficult to remove after treatment operation, which reduces their applicable viability. In this respect, nanofibers introduce a valuable substrate for the nano-powder, whether the nanoparticles are embedded into [21] or decorate [22] the nanofiber. This strategy keeps the nanoparticles separated, increases the nanoparticles’ surface area exposure, and increases the materials’ efficiency. Moreover, using nanofibers avoids a high cost and painful removal process. Previous strategies in modification of nanofibers, by adding function groups or adding nanoparticles, were seen to be effective in enhancing various properties (wettability, water contact angle, mechanical properties, cell adhesion and proliferation, adsorptive capacities, filtration, photocatalysis, catalytic degradation, etc.) of the nanofibers towards different applications.

Although there are a great many published reviews that represent the applications of electrospun nanofibers in particular fields, such as wastewater treatment [23,24,25,26,27,28,29,30,31], medical and biomedical applications [32,33,34,35,36,37,38,39,40,41], and other applications [42,43,44,45,46,47,48,49], and cover single or special topics of these fields, the reviews still need to be overviewed. Also, review articles that provide an overview and discuss more than one topic are very limited. Consequently, this article will review and highlight the most promising and recent research avenues for the application of electrospun nanofibers in some fields of biomedical science. Finally, recent achievements, challenges, and future perspectives of the application of electrospun nanofibers are pointed out. This work may guide scientists to overcome and cover the gaps in the scope of applications of electrospinning nanofibers in investigated fields of biomedical science.

## 2. Fabrication of Nanofibers via Electrospinning

**Figure 1:**
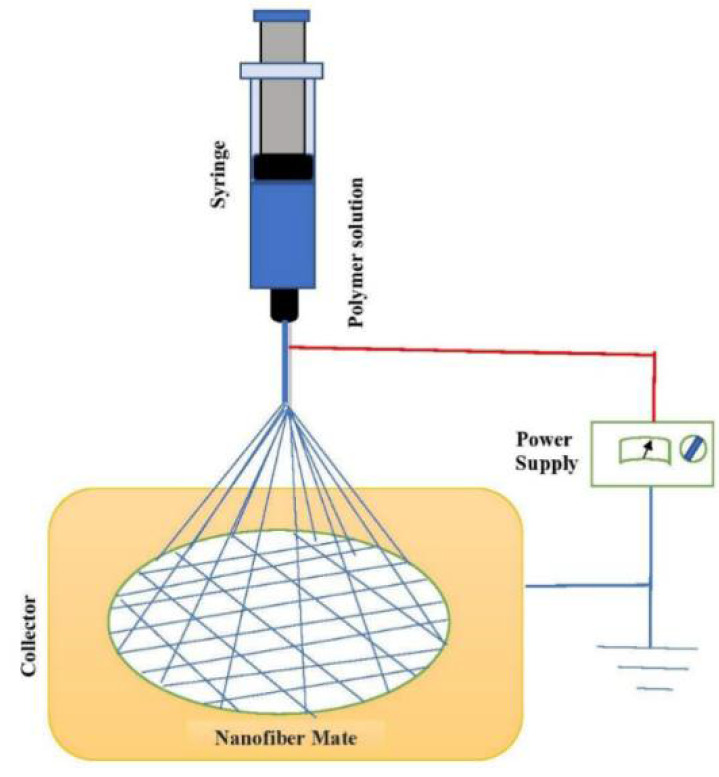
Schematic diagram for a conventional electrospinning process.

Electrospinning is a simple, cost-effective, and highly versatile technique used to fabricate a layer of nanofibers with diameters of 3.0–>5.0 nm, in the presence of a high-voltage electric field used on injected polymer solutions to stretch the droplets [43,50,51,52,53,54,55]. The high-voltage induces the interaction of charged polymer precursors and external electrical fields to form polymer nanofibers (PmNFs) [50], as in Figure 1. Where the high-voltage power source provides a high voltage of various tens of kilovolts [51] the fabricated nanofibers have unique characteristics, like higher surface areas and inter-/intra- fibrous porosity [4]. Therefore, it is used on a large scale to fabricate 1D continuous polymeric nanomaterials and 1D nanocomposites/inorganic nanomaterials [51].

Consequently, this technology has attracted great attention in fabricating different polymer ultrafine fibers [54]. Recently, the ability to fabricate new electrospun nanofibers and nanomaterials quickly by means of electrospinning technology led to the emergence of new applications in different fields [52]. To monitor the structures and alignment of these new electrospun nanofibers and nanomaterials, new strategies have been developed to open a world of opportunities in various applications [52]. Notably, many techniques have been developed to align the nanofibers. This confirms the feasibility of integrating the promising characteristics of fabricated nanofibers. Also, coaxial electrospinning was enhanced to manufacture continuous core-sheath and hollow nanofibers [52].

Recently, the fabrication of biomimetic scaffolds, using electrospun nanofibers, has attracted great attention regarding its use in tissue engineering applications. A densely packed 2D electrospun nanofibrous scaffold is considered a limitation impeding utilization in regeneration of tissues and organs [53]. These challenges have been overcome by developing simple and facile post-electrospinning procedures to adjust the densely packed 2D to low density 3D scaffolds, thereby enhancing their applicability in regeneration of tissues and organs [53].

Fabricated electrospun nanofibers have promising characteristics to use in biomedical applications, where they have the ability to be assembled in several shapes of 2D and 3D structures, with considerable surface/volume ratios, porosity, and adaptable pore sizes, and, furthermore, they can mimic fibers in a natural extracellular matrix.

According to the chemical composition of the fabricated nanofibers, they can be classified into four major types: 1—Inorganic nanofibers, 2—Carbon nanofibers, 3—Polymer-based nanofibers, 4—Composite nanofibers [56]. All of these types have potential application and functional performance in tissue engineering technologies, as discussed in the following sections. On the other hand, various types of nanofibers were recently fabricated from different organic and inorganic precursors, and the synthesis of nanofibers with distinguished morphological properties and satisfying yields is still challenging.

## 3. Applications of Nanofibers

During the last decade, electrospun nanofibers have attracted tremendous attention from researchers in materials science, nanotechnology, environmental science, and biomedical applications. Nanofibers’ superior characteristics and functions have attracted great attention and widespread application in different emerging fields. Their morphologies, chemical characteristics, and spatial distributions determine the potential application and functional performance [56,57]. Therefore, fabricated electrospun nanofibers used in medical applications must be safe and highly soluble.

### 3.1. Biomedical Applications

Due to the efficiency and promising properties of nanofibers in the treatment and curing of medical complications, like bone cell proliferation, vascular scaffolds, and skin tissue engineering, recent developments in nanofibers have made great progress in biomedical fields [39,57]. Consequently, various novel nanofibrous products for biomedical applications have been studied and investigated. The morphologies, fiber diameters, and homogeneity of fabricated electrospun nanofibers are monitored by parameters controlled by the electrospinning processes [57].

#### 3.1.1. Bone Cell Proliferation

Bone cell proliferation processes are complicated and have many defects where self-regeneration is restricted. Therefore, scaffolds are widely utilized in replacement and regeneration processes for damaged bone tissues. During the last decade, various types of bionanomaterials have been used to design porous scaffolds that mimic the structure of the original ECM [58,59,60]. Thus, many works have been investigated to fabricate scaffolds.

To culture the bone marrow endothelial progenitor cells (BEPCs), two varied hybrid scaffolds, composed of collagen/polycaprolactone (PCL) (70:30%) and gelatin (Gel)/PCL (70:30%), were prepared by electrospinning [58]. BEPCs were separately seeded on the two scaffolds and on glass slides as a control group. BEPCs spread well and adhered strongly to the collagen/PCL and gel/PCL scaffolds, compared with the control group (glass slide). Moreover, the expression of inflammatory factors, containing interleukin (IL)-1, showed a high decrease on the gelatin (gel)/PCL scaffold compared with the collagen/PCL scaffold and the control group. Electrospun collagen/PCL and gelatin/PCL scaffolds presented potential to improve the adherence and proliferation of BEPCs [58]. On the other hand, dual non-woven ultrafine nanofibrous scaffolds, consisting of polyurethane (PU)/Nylon 6 (N6) hybrid polymers, blended with natural gelatin (Gel), were prepared by a dual syringe electrospinning approach. Biomimetic manner characteristics seen at various time intervals exhibited the construction of a bone apatite-like layer on the dual nanocomposite nanofibrous scaffolds, proving significant bioactivity. The dual (PU-Gel)/(N6-Gel) nanocomposite nanofibrous scaffolds have high wettability, supporting excellent osteoblast cell proliferation [59].

Recently, gelatin nanofibers have acquired considerable attention due to their promising characteristics as eco-friendly materials and they have considerable influences on cell adhesion, proliferation, and differentiation of diverse tissues [60]. Glucose cross-linked gel/zein scaffolds were evaluated to regenerate bone in vivo and in vitro. The nanofiber scaffolds presented rapid mineralization in the concentrated mimicked body fluid with precipitated octacalcium phosphate, and dicalcium phosphate dehydrate. Cytotoxic effect on MC3T3e1 cells in a CCK-8 test of the nanofibrous scaffolds was negligible. Osteogenesis characterizations were tested with Alizarin Red staining and showed enhanced calcium precipitation on the cross-linked scaffolds, while the alkaline phosphatase (ALP) exhibited no variation.

Moreover, the in vivo cranial bone regeneration investigation found that cross-linked gel/zein scaffolds showed high positive effects on the regeneration of cranial bone with enhanced new bone volume and connective tissue formation. On the other hand, the presence of zein in the gelatin scaffolds did not favor the regeneration mechanism. Furthermore, cross-linking of gelatin scaffold decreases bone resorption, which hinders osteoclasts’ differentiation [60]. Also, polyd,l-lactide, gelatin, and RKKP glass-ceramics were used to form a tricomponent electrospun scaffold. The bioactivity RKKP glass-ceramic system arises from La^3+^ and Ta^5+^ ions, which act as growth supporting agents for bone. RKKP glass-ceramics have encapsulated the microfibers of electrospun scaffolds, and the results confirm its homogenous distribution in the fibrous composite.

Moreover, glass-ceramics allowed the biomineralization of the scaffolds and enhanced cell viability and osteogenic differentiation. The mineralized layer, composed of RKKP-including scaffolds after soaking in a simulated body fluid medium, is hydroxyapatite as characterized by different characterization techniques. The results of differentiation investigations of canine adipose-derived mesenchymal stem cells grown on the electrospun scaffolds suppose that changing the amount of RKKP in the scaffolds can drive differentiation toward differentiation chondrogenic or osteogenic commitment. The existence of ions, such La^3+^ and Ta^5+^, in the RKKP incorporated polymeric nanocomposite scaffolds could improve cell growth and allow differentiation [61].

Structures of many natural materials, such as chitosan and its bioactive polymers, mimic exceedingly well to glycosaminoglycan (GAG). GAG is one of the components of bone extracellular matrix (ECM), which plays a vital function in cell-cell adhesion by interaction with collagen fibers [62,63,64,65]. These biomaterials have good biodegradability, biocompatibility, suitable mechanical properties, easy processing ability, water solubility, and can chelate with Ca or other active components during biomineralization processes [63,64]. Such biomaterials have attracted great attention during recent years in biomedical applications. Therefore, as a solvent, chitosan (Chi) nanofibers were electrospun from aqueous chitosan solutions via concentrated solutions of acetic acid (CH_3_COOH). Polyethylene oxide (PEO), of different weights (10–60 wt%), was blended with Chi solutions which acted as a plasticizer to enable spinnability of the Chi solutions that formed. MTT-assay and ALP expression analysis shows that it reached eleven days of cell seed. The fabricated electrospun Chi scaffolds developed MG 63 cell proliferation and differentiation into mature osteoblasts [62]. Also, metformin-loaded polycaprolactone/chitosan nanofibrous membranes were prepared using an electrospinning strategy for bone repairing membranes with better osteoinductive properties, subsequently espoused to glutaraldehyde crosslinking to improve the stability of chitosan in aqueous media. Furthermore, rats’ bone mesenchymal stem cells were seeded on membranes for the evaluation of the effect of metformin-loaded polycaprolactone/chitosan nanofibrous membranes on cell morphology, alkaline phosphate activity, and osteogenic mineralization in vitro. Moreover, in vitro experiments supposed that the crosslinked-polycaprolactone/chitosan/metformin membranes provide a preferable environment for cell attachment, proliferation, and osteogenic differentiation of bone mesenchymal stem cells [63]. Also, electrospun PCL/carboxymethyl chitosan (PCL/CMChi) nanofibers cured by helium cold atmospheric plasma (CAP) and incubated with bone morphogenic protein-2 (BMP-2) acted as scaffolds for the osteodifferentiation of stem cells. For the in vitro test, human bone marrow-derived mesenchymal stem cells (hMSCs) were seeded on the fabricated scaffolds, and their activities were followed. The findings exhibited that scaffolds supported the proliferation of hMSCs and enhanced their osteodifferentiation without using additional osteogenic differential agents. Moreover, the RT-PCR and ICC results indicated that CAP treatment and BMP-2-fmodifications exhibit synergic improvement on the ossification of hMSCs [64].

Due to the physicochemical characteristics and biocompatibility of electrospun collagen-chitosan membranes, and their role in guided bone regeneration, electrospinning techniques were used to fabricate electrospun collagen matrices and electrospun collagen-Chi matrices. In vivo, calvarial bone defects generated on rats were coated with two various types of membranes, respectively. The results indicate that regular and highly-porous membrane was required for excellent attachment and proliferation of periodontal ligament cells which was seen in all electrospinning mates. Additionally, electrospun collagen-Chi matrices showed excellent physiochemical characteristics, such as high tensile strength and more biodegradation stability rates, compared with electrospun collagen membranes. In the animal model, electrospun collagen-Chi membranes presented enhanced bone ALP levels in the fourth week and osteocalcin in the eighth week, compared with other groups. Moreover, both the radiographical and histological analyses further indicated that electrospun collagen-chitosan nanofiber donated high performance in the formation of new bone [65].

**Figure 2:**
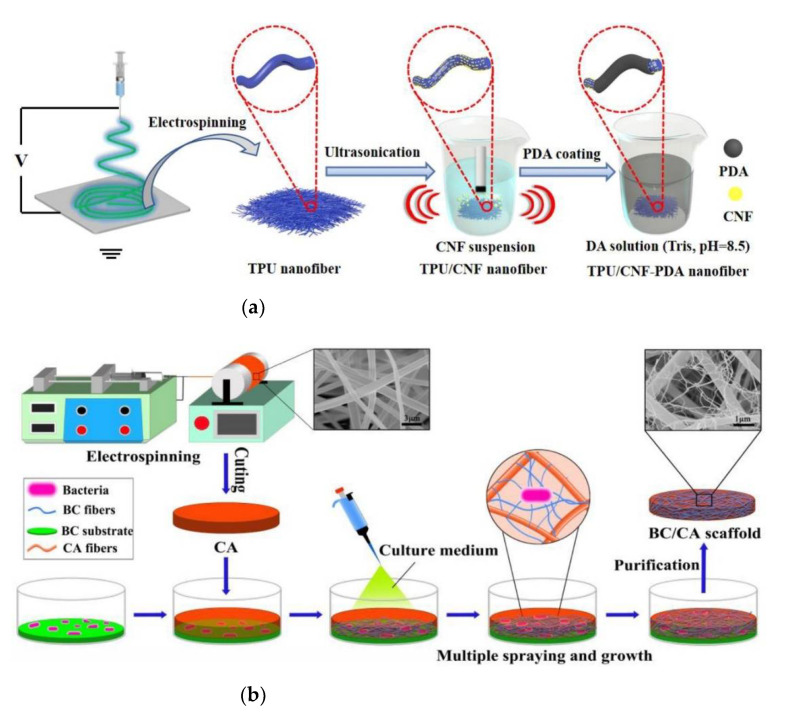

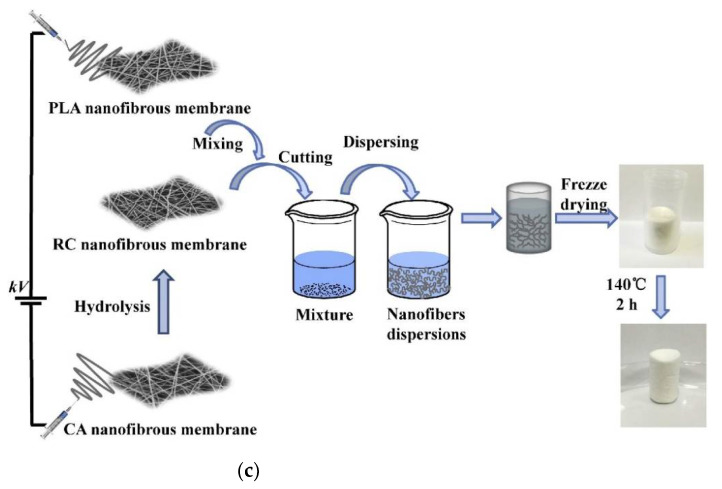
Schematic diagram of preparation of (**a**) TPU/CNF-PDA nanocomposite nanofibers (reproduced from [66] with permission of Taylor & Francis, 2019), (**b**) nano-submicron BC/CA scaffold, CA sub-microfibrous scaffold was fabricated by electrospinning and BC nanofibrous scaffold by in situ biosynthesis. BC/CA scaffold was prepared by spraying culture medium into the CA scaffold located on top of the BC base film, followed by multi-step in situ biosynthesis inside the CA scaffold (reproduced from [67] with permission of Elsevier, 2019), and (**c**) PLA/RC scaffold fabrication (reproduced from [68] with permission of Elsevier, 2019).

Recently, various biomaterials, such as cellulose (C), were investigated and introduced to improve interactions between cells and scaffolds. Thermoplastic polyurethane (TPU) nanofibers were prepared via an electrospinning strategy, and then functionalization of the surface with cellulose nanofibril (CNF) particles by means of ultrasonic assistance was conducted to produce TPU/CNF nanofibers. Thereafter, they were coated with polydopamine (PDA) to core/shell structure of TPU/CNF- (PDA nanocomposite nanofibers. Compared to thermoplastic polyurethane nanofibers, the homogeneity of the polydopamine anchoring layer on the TPU/CNF nanocomposite nanofibers surface highly increased attributes to the introducing of CNF which acted as the active binding sites to induce the polydopamine particles to deposit along with the nanofibers. The swelling and hydrophilicity properties of TPU/CNF-PDA composite nanofibers were enhanced, compared with those of TPU and TPU/CNF nanofibers. The schematic diagram for the fabrication of TPU/CNF-PDA nanocomposite nanofibers is illustrated in Figure 2a. Moreover, the TPU/CNF-PDA nanocomposite nanofibers provide mechanical properties higher than those of the TPU and TPU/CNF nanofibers, this is in reference to the formation of strong H-bonds between PDA and TPU/CNF, making TPU, CNF and PDA strongly attached to each other. The adhesion and viability of mouse embryonic osteoblast cells (MC3T3-E1) seeded on TPU/CNF-PDA nanocomposite nanofibers were clearly developed, in comparison with TPU and TPU/CNF nanofibers [66].

Bacterial cellulose (BC) nanofibers were incorporated with CA sub-microfibers to simulate the fibrillar design of natural ECM. The blending of CA sub-microfibers with BC nanofibers caused enhancement in mechanical properties as well as in porosity of the scaffold. Additionally, the biological properties of investigated BC/CA on MC3T3-E1 demonstrated that BC/CA scaffolds enforced cell migration and proliferation, as represented in Figure 2b. Furthermore, the BC/CA scaffolds exhibited great alkaline phosphatase (ALP) activities, and calcium depositions [67]. Also, the hierarchical designs highly enhanced the expression of the osteogenic gene (ALP mRNA and Runx2 mRNA) and protein (ALP) [67].

On the other hand, freeze-drying and crosslinking approaches were utilized to form a 3D polylactic acid/regenerated carbon (PLA/RC) scaffold. The addition of RC nanofibers to the scaffold improved its hydrophilicity and biological performance. Moreover, citric acid was added, as a green crosslinker, to form esterification-crosslinking interaction with RC nanofibers. Furthermore, unincorporated −COOH groups can also contribute to 3D scaffolds with apatite nucleating capacity for further boosting of osteogenic potential. Prepared PLA/RC nanofiber-reformed scaffolds, shown in Figure 2c, show enhanced swelling of the characteristics, hierarchical cellular design and rapid removal from the 80% strain. Obviously, well-structured PLA/RC scaffolds, decorated with –OH and –COOH groups, presented high biomineralization activity in the SBF solution. Additionally, the formation of bonelike apatite can retard acid degradation products from PLA, and will also reinforce scaffold-to-bone interaction in the implantation process [68]. Also, electrically conductive nanofiber scaffolds, composed of polyaniline-co-(polydopamine-grafted-poly(d,l-lactide)) [PANI-co-(PDA-g-PLA)], were prepared by an electrospinning strategy for bone tissue engineering. In detail, PANI-co-PDA was prepared through a one-step chemical oxidization technique. After that, d,l-lactaide monomer was blended onto the PDA part via a ring-opening polymerization (ROP) to form PANI-co-(PDA-g-PLA) terpolymer. Finally, a solution of the prepared terpolymer was electrospun to form a conductive nanofiber scaffold. The experimental results showed enhancement in physicochemical properties, such as mechanical properties, conductivity, electroactivity, hydrophobicity, and morphology, as well as biological features involving biocompatibility, biodegradability, and improvement in the cells’ attachment and proliferation [69].

Bovine serum albumin (BSA) is a small molecules protein and is considered to be promising material with excellent water solubility, and is non-expensive. Therefore, it is utilized in pharmaceutical industries and tissue engineering as a protective agent to several growth factors [70]. So, poly l-lactic acid-co-e-caprolactone (PLCL) beaded nanofiber with core-shell morphology (130 ± 30 nm) was synthesized, and bovine serum albumin was incorporated into the inner layer. The obtained results from in vitro cultivation of human mesenchymal stem cells (hMSCs) on the (PLCL/BSA) core-shell beaded nanofibers’ surfaces supposed that the beaded nanofibers, with core-shell structure, could highly promote adhesion and proliferation of cells [70].

The biodegradability of polymers is a significant factor that controls the utilization of these materials as reservoirs to modulate release kinetics of proteins and small molecules [71]. Therefore, a polylactic acid electrospun nanofiber shell incorporating platelet-derived growth factor (PDGF-BB) was investigated. The release of PDGF-BB was controlled by introducing water-soluble polyethylene glycol to the polylactic acid shell to act as a porogen. The core-shell nanofibers created 3D scaffolds with an internal macroporous design, with suitable mechanical functions and with high cell compatibility. Introducing PDGF-BB enhanced cell viability, proliferation, and penetration, and upregulated key genes included in the meniscal extracellular matrix (ECM) formed in human meniscal and synovial cells [71]. Also, various types of hybrid scaffolds are utilized to overcome the limitations and deficiencies of scaffolds fabricated from material blends [72]. To achieve these, scaffolds were composed from 3D freeze-dried gelatin and electrospun poly(lactide-co-glicolide) (PLGA) fibers in a ratio of one to one (*w*/*w*). To enhance the osteoblast proliferation of the scaffold, hydroxyapatite nanoparticles (HA) anchored the fibers employing a sonochemical approach, followed by crosslinking using EDC/NHS solution. Swelling and TGA assessment confirmed the hybrid scaffolds possess a higher crosslinking degree than a pure gelatin scaffold, resulting from covalent interactions among gelatin, polylactide-co-glicolide, and hydroxyapatite nanoparticles. In addition, 3D hybrid scaffolds possessed mechanical properties higher than individual hydrogels. In vitro experiments provided good fibroblast and osteoblast proliferation and migration on the 3D hybrid scaffolds, and they also deeply moved through their structures during the one week of the test [72]. On the other hand, an electrospinning strategy was investigated to synthesize core–shell nanofibers, where alginate was cored in the shell of a water-insoluble polymer and aligned nanofibers were collected with the utilization of a high-rotational speed collector. The alginate was crosslinked with calcium ions and subsequently washed to remove the shell polymer. By loading fibronectin on the prepared alginate fibers, and by seeding the hMSCs cells, cells can be elongated in the fiber direction [73].

**Figure 3:**
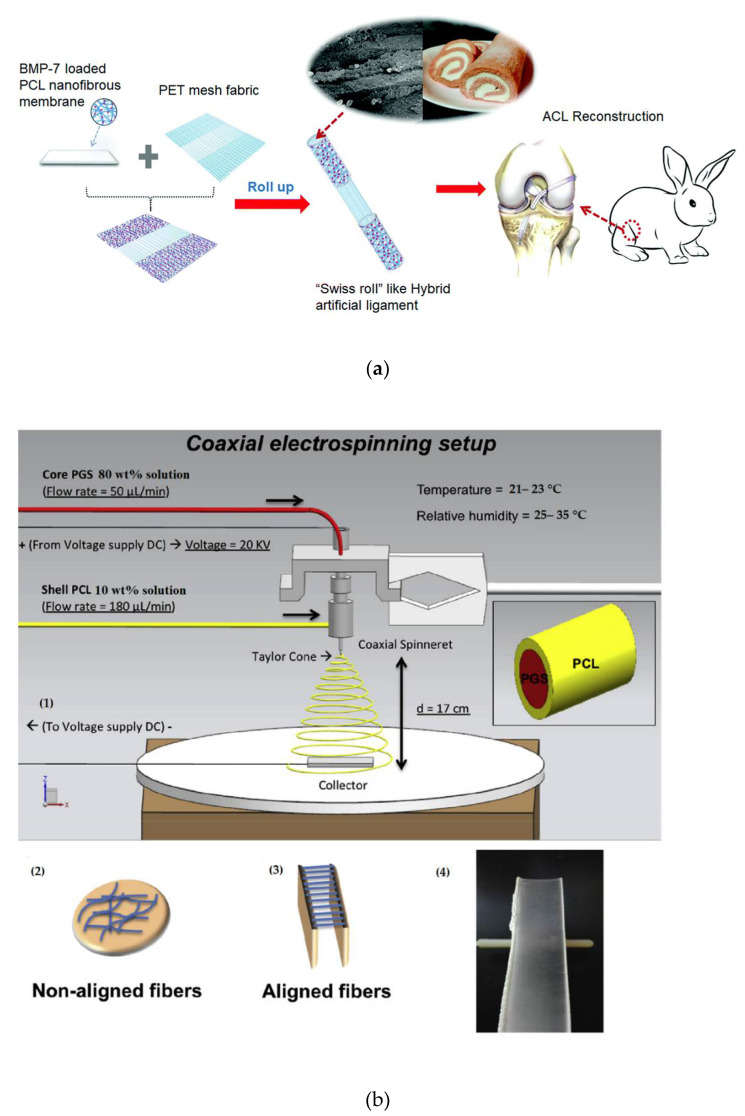
Schematic diagram of fabrication of (**a**) novel hybrid artificial ligament used for reaching the “ligamentization” after ACL reconstruction (reproduced from [74] with permission of Royal Society of Chemistry, 2019). (**b**) coaxial aligned PGS/PCL nanofibers (1). Non-aligned nanofibers were produced in a round copper collector plate (2), and aligned nanofibers were recovered in a two parallel copper plate collector (3, 4) (reproduced from [75] with permission of Elsevier, 2019).

In sports medicine, the fabrication of a novel hybrid ligament for anterior cruciate ligament (ACL) reconstruction contains biodegradable scaffolds with polyethylene terephthalate (PET) nanofabrics to solve the deficiencies of classical degradable and nondegradable scaffolds [74]. Therefore, novel bone morphogenetic protein 7 (BMP-7)-immoblized (PCL) nanofiber membranes were prepared using layer-by-layer (LbL) self-assembly. Subsequently, multifunctional hybrid ligaments were made by rolling up the nanofiber materials and polyethylene terephthalate (PET) mesh fabric (nondegradable part) into a “swiss roll” structure, as shown in Figure 3a. The in vitro studies showed that the hybrid ligament could enhance the biocompatibility of the pure polyethylene terephthalate ligament and further support the mineralization of cells. The in vivo tests indicated that this superior design significantly enhanced the combination of hybrid ligaments and bone tunnels, thereby constructing real “ligamentization” after anterior cruciate ligament reconstruction surgery [74]. Another work was investigated to improve the coaxial of poly glycerol sebacate (PGS), as the core, with PCL as the sheath, of electrospun aligned-nanofibers, able to enhance a sustained release of Kartogenin (KGN) while being compatible with the size and alignment of native articular cartilage extracellular matrix (ECM) [75].

In this work, a coaxial electrospinning of PGS)/(PCL aligned nanofibers (core:PGS/shell:PCL) was investigated. Different properties of aligned coaxial nanofibers, aligned monoaxial PCL fibers and non-aligned controls were compared, as represented in Figure 3b. All the resulted electrospun scaffolds had fibers with diameters within the nanometer-scale range. The core-shell morphology of the nanocomposite nanofibers was indicated by TEM. In addition, alignment of the fiber highly induces elastic modulus by >2-fold for both coaxial and monoxial scaffolds. Furthermore, KGN, which is a small molecule known to support mesenchymal stem/stromal cells (MSC) chondrogenesis, was immoblized into the core polyglycerol sebacate solution to fabricate coaxial PGS-KGN/PCL nanofibers to support MSC chondrogenesis. The release kinetics of studies of KGN and biological activity of the scaffold were compared to KGN-assembled monoaxial fibers and non-loaded controls. The results indicated that the coaxial nanofibers (PGS-KGN/PCL) can release KGN over 21 days with more control than monoaxial PCL-KGN nanofibers. Moreover, KGN-loaded scaffolds highly increased MSC cell proliferation and chondrogenic differentiation, as expected by the increased sGAG content and chondrogenic marker gene expression levels [75].

Over the last years, various electrical stimulation techniques have been applied to enhance bone regeneration and fracture healing. Though direct current field (DCF) stimulation has many advantages, compared to other approaches, it has many drawbacks, such as the possibility of releasing electrochemical materials and wear debris from the inserted electrodes [76]. Therefore, electroconductive scaffolds-medicated-DCF are one of the most important materials used to overcome the afore-mentioned defects [76]. Electroconductive electrospun carbon nanofibers (CNFs) were prepared to apply bone cell electrical stimulation. The CNFs were fabricated using electrospun polyacrylonitrile (PAN) nanofibers via two steps, stabilization and carbonization. The cultured CNFs with Mg-63 cells (SCNFs) were subjected to DC electrical fields with current intensities of 10, 50, 100, and 200 μA. The findings showed that growth of the cultured cells was highly enhanced on connection with the DC electric field and caused the best proliferation level, 116.43 ± 4.76%, at 100 μA. The alkaline phosphatase (ALP) performance analysis exhibited increased osteogenic activity of cells, required for the bone healing process, as a result of the applied field [76].

On the other hand, alveolar bone loss is always a major challenge that faces oral implant placement and guided bone regeneration (GBR) is considered to be a promising solution to these issues. Among all the factors, the barrier membrane plays a vital role in these technologies. Till now, lack of osteoinductivity has still been the biggest weakness for clinical applications. Therefore, many works have been done to fabricate osteoinductive three-dimensional (3D) nanofiber membranes, fabricated with a drug releasing system by adding parathyroid hormone (PTH)-Fc into the poly l-lactic acid-co-e-caprolactone (PLCL/SF) solutions for electrospinning. The results showed that the 3D nanofibers have appropriate physical and chemical function and good biocompatibility. The effect on the bone marrow mesenchymal stem cells (BMMSCs) showed the drug release supporting osteogenic differentiation of cells stably [77].

3D printed biodegradable calcium phosphate scaffolds with antibacterial activity are one of the main requirements for repairing of jaw bone. Calcium phosphate powder and berberine were mixed to modulate the printing inks. Porous scaffolds were prepared by direct extrusion 3D printing and cross-linked with sodium alginate in situ. The release of the antimicrobial drug, berberine, is controlled by adjusting the cross-linking degree of the scaffold. The young’s modulus of 3DP scaffolds was recorded at about 1.3 MPa. After freeze-drying, the 3DP scaffolds were shrunk by 24.4% for less swelling, confirming the suitable structure stability of the scaffold. In vitro biological studies exhibited that the 3DP scaffolds had soft cytotoxicity and it enforced MC3T3 cell attachment and proliferation [78].

**Figure 4:**
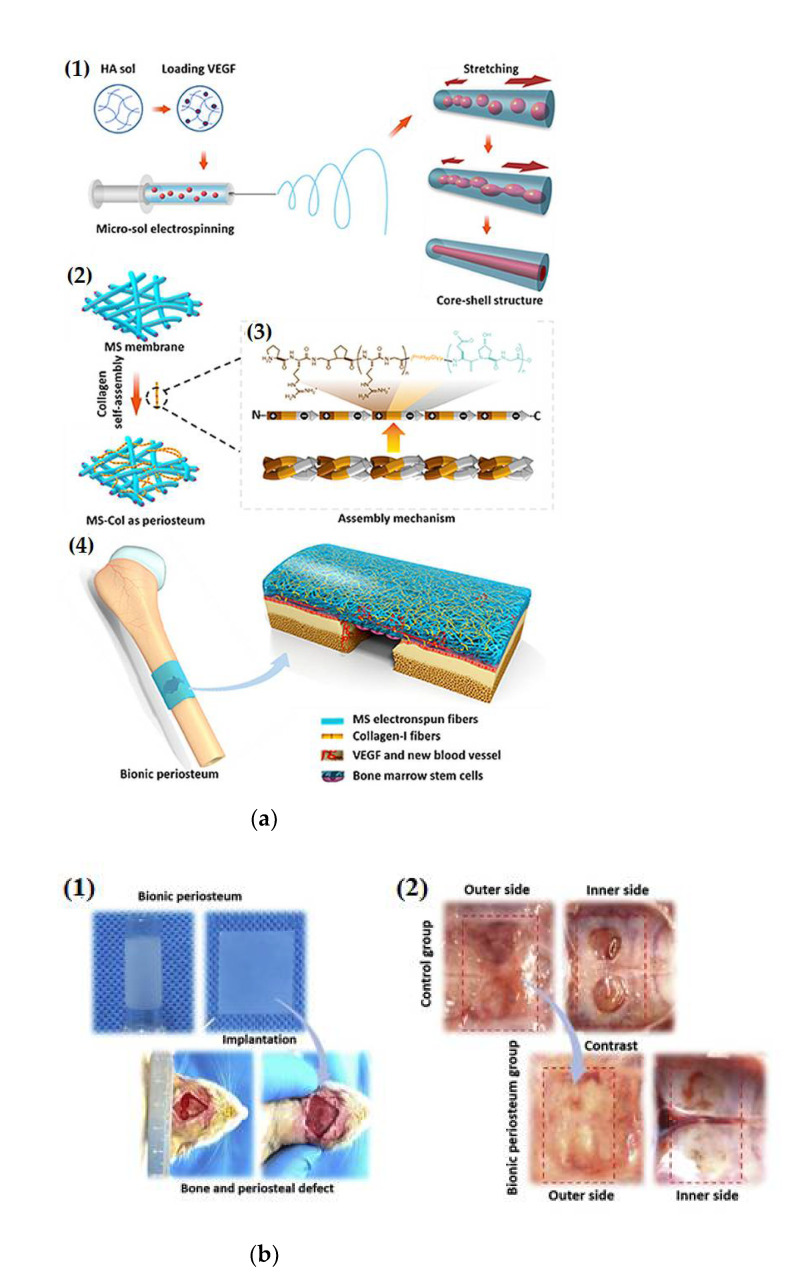
Schematic illustration (**a**) (1) The fabrication of VEGF-loaded electrospinning membranes by hyaluronan (HA) micro-sol electrospinning and the mechanism of formation of core-shell structure. (2) The building process of the hierarchical micro/nanofibrous structure along with (3) the illustration of assembly mechanism and (4) bionic periosteum for periosteal and bone regeneration, and (**b**) General evaluation of in vivo performance. (1) Images represent the critical bone and periosteal defect model and implantation of bionic periosteum with good operability, and (2) Images represent the contrast between bionic periosteum and control group from the outer and inner side of skull specimens (reproduced from [81] with permission of Elsevier, 2019).

In many fabrication approaches, glacial acetic acid (GAC) is used as a solvent of polycaprolactone to prepare microfibers or beaded fibers. To overcome this defect, a nontoxic assistant solvent, ethylene carbonate (EC), was utilized in the PCL/GAC system to successfully prepare electrospun nanofiber (PCL) scaffolds. The diameter of PCL fiber reduced with further increase in EC0 concentration in range 0–9 vol% and began to slightly increase beyond 9 vol% of EC. MTT results demonstrated that MC3T3-E1 cells on the PCL scaffolds showed development on cell proliferation [79]. Two different three-dimensional (3D) scaffolds, polytetrafluoroethylene (PTFE), and polyvinyl alcohol (PVA), were used to form PTFE/PVA polymers with and without graphene oxide (GO) nanoparticles. After synthesizing the (GO)NPs, two types of 3D scaffolds, PTFE/PVA (PP) and PTFE/PVA/GO (PPG), were fabricated, employing chemical cross-linking and freeze-drying techniques for bone tissue regeneration. The two 3D scaffold types had nanotopographical designs, excellent porosities, hydrophilic features, thermal stabilities, and high stiffness, and promoted cell adhesion, proliferation, and osteogenic differentiation. Importantly, GO blended scaffolds revealed a better milieu for cell manners [80]. A hierarchical micro/nanofibrous bionic periosteum was fabricated with controlled releasing of VEGF as an exogenous vascularized fibrous layer of periosteum to encourage an endogenous cambium layer in vivo for complete restructuring of periosteal and bone tissue, via collagen self-loading and micro-sol electrospinning techniques, as illustrated in Figure 4a, [81]. The VEGF incorporated hyaluronan-PLLA core-shell design was introduced to be released in a sustainable manner for angiogenesis in the fibrous layer and bone-cured area. Meanwhile, the self-loading of collagen combined with electrospun fibers shared a hierarchical micro/nanostructure which highly simulated the microenvironment of extracellular matrix to present structural and biochemical cues for cell adhesion, proliferation and differentiation, and caused the production of a cambium layer, which simulated the in-situ ossification as intramembranous ossification. The periosteal biomaterial results showed unique activity of scar inhibition, angiogenesis, and osteogenesis to remodel the bone defect in a uniform and fast way by an inherent periosteal ossific mechanism, included in both intramembranous and endochondral ossification [81]. The evaluative study of in vivo performance is shown in Figure 4b.

Due to shortage of considerable bioactive biomaterial scaffolds, effective disease-induced or critical size bone regeneration is still a challenge in tissue engineering [82]. Therefore, the fabrication of bioactive nanofibrous scaffolds have attracted great attention from many more researchers recently [83]. Bioactive nanofibrous scaffolds are prepared by spinning a blend of PCL, elastomeric poly citrates-siloxane (PCS), and bioactive osteogenic miRNA nanocomplexes (denoted PPM nanofibrous scaffolds) with controlled miRNA release, synthesized for supporting bone regeneration depending on combination of a physico-chemical-biological approach. The PPM scaffolds showed homogenous nanostructures, improved tensile stress (≈15 MPa) and modulus (≈32 MPa), enhanced hydrophilicity (30–60°), gentle biodegradation, and high blue fluorescence. Bioactive miRNA complex is highly assembled into the nanofibrous matrix and presents long-term release over seventy hours. The PPM scaffolds greatly promote the attachment, proliferation, and osteoblast differentiation of bone marrow stem cells in vitro and improved rat cranial defect recovery (twelve weeks) in vivo [82].

To reduce the pain and inflammation of joints resulting from bone fractures, various biomaterials, such as ghee, banana, turmeric, dried ginger and castor oil, were investigated [83]. In many cases, pure ghee is used as one of the components to treat pain due to bone fracture. Novel PU/ghee/propolis nanofibrous composite was investigated via the electrospinning strategy. The results indicated that the fabricated electrospun scaffolds showed excellent blood compatibility, with non-hemolytic properties, improved safety to RBCs and had no toxicity behavior. Therefore, PU/ghee and PU/ghee/propolis nanocomposites could be presented as a suitable candidate for bone tissue repairing [83]. Furthermore, different polylactic-co-glycolic acid (PLGA)-based biomaterials are promising nanoparticles in bone tissue engineering applications [84]. Different amounts of PLGA/hydroxyapatite (HA) nanofibers assembled with dexamethasone (Dex) were fabricated using the electrospinning approach. It was found that 0.5% (wt) Dex in (HA) scaffolds were sufficient for anti-inflammatory effects. Admittedly, dexamethasone had some cytotoxic effect on osteoblasts and an inhibitory effect on alkaline phosphatase activity. On the other hand, the relatively low dexamethasone (<2% [wt]) had no inhibitory effect on osteoblast maturation and mineralization. By this token, dexamethasone is considered aed candidate to improve biocompatibilities of polylactic-co-glycolic acid-based bionanomaterials. However, the cytotoxic effect of Dex should be of concern [84].

Generally, natural bone has innate capacities to reconstruct after damage. Nevertheless, massive bone problems resulting from injury, resection, and disease pose an enormous challenge to healing surgery. Therefore, reconstructed damaged bone is a great goal to reduce pain and speed up reconstitution of damaged bone. So, nanofibrous scaffolds fabricated via the electrospinning approach need to provide mechanical characteristics which can withstand cell cultivation to a definite period and upkeep of cell segregation. To overcome such challenges, novel biomaterials and nanofibrous scaffolds are needed to develop and fabricate safe, effective, and smart electrospun nanofibers with desired outstanding electrical, mechanical, and nanofibrous structural characteristics. Also, interdisciplinary collaboration of scientists and researchers from various fields must continue to develop the field of biomaterials and help tackle the challenges that lie ahead. As well, concerted efforts must be made to transfer such novel biomaterials from the laboratories into clinical practices where continuous technological advances offer promising solutions for many challenges.

#### 3.1.2. Nerve Regeneration

**Figure 5:**
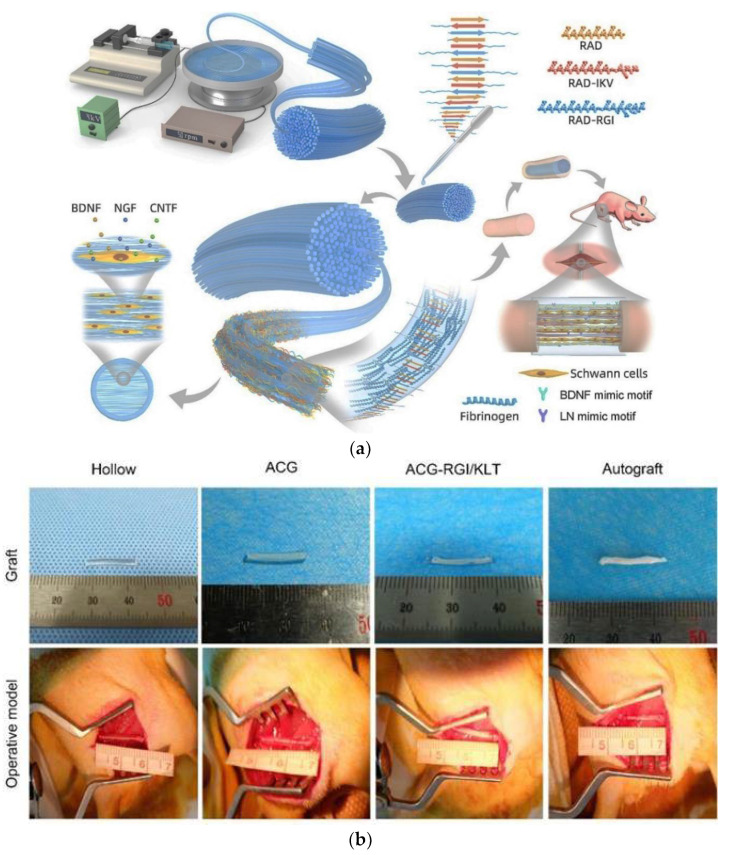
(**a**) Schematic diagram of the fabrication of AFG/fSAP interpenetrating hydrogel and the in vitro and in vivo evaluations (reproduced from [85] with permission of Elsevier, 2021), and (**b**) Gross view of nerve grafts and operative model of all groups. The chitin conduits were light yellow in color and transparent. ACG and ACG-RGI/KLT were white, similar to sciatic nerves. All groups received the 15-mm sciatic nerve defect (reproduced from [87] with permission of KeAi Publishing, 2020).

Peripheral nerve injuries (PNI), caused by traffic accidents, natural disasters, and unsatisfactory treatments [85], are intractable clinical problems that create heavy burdens for patients. Therefore, various electrospinning nanofiber materials play a considerable role in nerve regeneration. So, an aligned fibrin/functionalized self-assembling peptide (AFG/fSAP) interpenetrating hydrogel was fabricated by electrospinning and molecular self-assembly, where the prepared hydrogels show synergistic topographical and biochemical cues [85]. The scaffolds with aligned structures illustrated considerable impact on the regeneration of peripheral nerves [85]. Also, they upregulated regeneration-associated gene expression and activated PI3K/Akt and MAPK signaling pathways in regenerated nerves. Figure 5a explains the synthesis of AFG/fSAP interpenetrating hydrogel and the in vitro and in vivo evaluations. Also, the influence of carbon nanofibers (CNFs) suspended PCL nanocomposite coatings and biomolecules loading on silk fibroin (SF)-based conducting braided nerve conduits was examined for improving Neuro 2a cellular activities. A superior conjugation of biomolecules (SCM) and various ratios of carbon nanofibers (5, 7.5, 10% *w*/*w*) were suspended in 10% (*w*/*v*) polycaprolactone solution to anchor on degummed silk threads. The loaded silk threads were braided to improve the scaffold structures. Increase in the carbon nanofiber concentration in the coating material leads to enhanced conductivity, tensile and mechanical features. In vitro cytocompatibility studies showed that the braided conduits were non-toxic. Cell adhesion, culture and proliferation were highly promoted on the biomolecule modified nanocomposites loaded silk braided structures, indicating their activity in regenerating and recovering peripheral nerve tissue [86].

Aligned chitosan nanofiber hydrogel (AChiG) incorporated with a bioactive peptide mixture composed of Ac-RGIDKRHWNSQGG (RGI) and Ac-KLTWQELYQLKYKGIGG (KLT), named AChiG-RGI/KLT, was applied as nerve conduit filler to remodel sciatic nerve defects in rats. AChiG-RGI/KLT oriented the Schwann cells, and supported the proliferation and excretion of neurotrophic factors by Schwann cells. At an early infection stage, AChiG-RGI/KLT promoted nerve regeneration and enhanced vascular infiltration. After twelve weeks, AChiG-RGI/KLT facilitated nerve repair and functional restoration in rats [87]. The nerve stumps were bridged with nerve grafts as shown in Figure 5b.

Generally, nanofibrous scaffolds in nerve regeneration need many requirements, such as guided axons, appropriate strength to resist tensile movements, and favorable electric conductivities to promote adhesion of cells, proliferation, and infiltration so as to solve many challenges, like secondary surgery and neuroma formation. Also, the development of artificial scaffolds that can mimic the natural extracellular matrix (ECM) is another challenge. Therefore, such biocompatible scaffolds, embedded with nerve cells and synthetic functional components, can be implanted as a conduit for axonal regenerations and facilitation of renewed nerves.

#### 3.1.3. Vascular Tissue

Recently, electrospinning has been utilized to synthesize nanofiber-based scaffolds. Several works have investigated and described the applications of nanofiber-based scaffolds for vascular scaffolds of small diameter and vascular grafts [88,89,90,91,92,93,94]. Though the vascular scaffolds fabricated by present electrospinning techniques can mimic the compositions of human blood vessels, these technologies have many difficulties in fabrication of vascular scaffolds of small-diameters (<1.5 mm) [88]. Therefore, a biodegradable polyl-lactide-co-caprolactone (PLCL) with biomimetic mechanical characteristics was employed to fabricate small diameter <1.5 mm PLCL/tussah silk fibroin (TSF) nanofiber vascular scaffolds for grafting. The biological behaviors of PLCL/TSF nanofiber vascular scaffolds were tested by in vitro culture of vascular endothelial cells (ECs). The scaffolds efficiently allowed vascular endothelial cell adhesion and proliferation [88]. Also, to mimic and fabricate the specific structures of natural blood vessels, a new approach has been developed [89]. In this process dual-oriented/bilayered small-diameter tubular nanofiber scaffolds were prepared by a mixture of PCL, poly d,l-lactide-co-glycolide (PLGA) and gelatin. The two bilayered nanofibers were orientated perpendicular to each other, aiming at guiding cell-specific orientation of smooth muscle cells (SMCs) and endothelial cells (ECs) in vitro, respectively. The findings revealed that the presence of gelatin highly induced the hydrophilicity of the scaffold as well as its mechanical property. The in vitro degradation indicated that by blending of three biodegradable polymers, the degradation rate of the scaffold accelerated. Moreover, electrospun scaffolds could enhance proliferation of both SMCs and ECs. Furthermore, topographic cues signed by oriented nanofibers could direct the growth and orientation of smooth muscle cells and endothelial cells [89].

**Figure 6:**
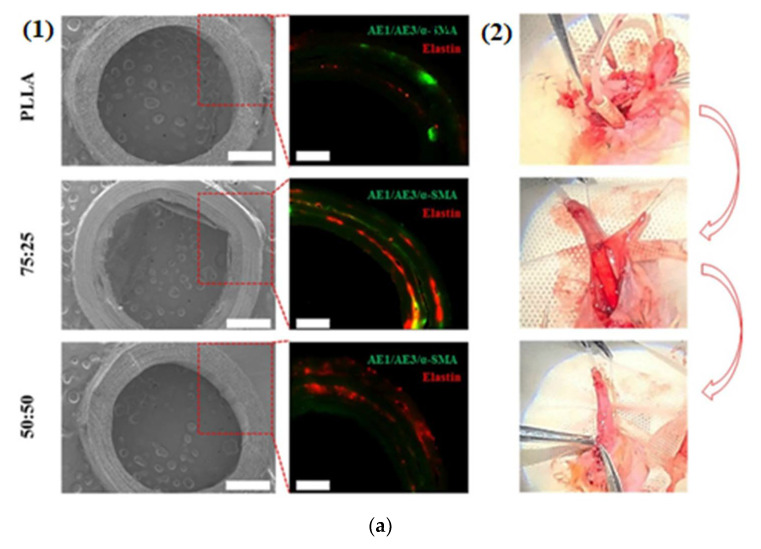

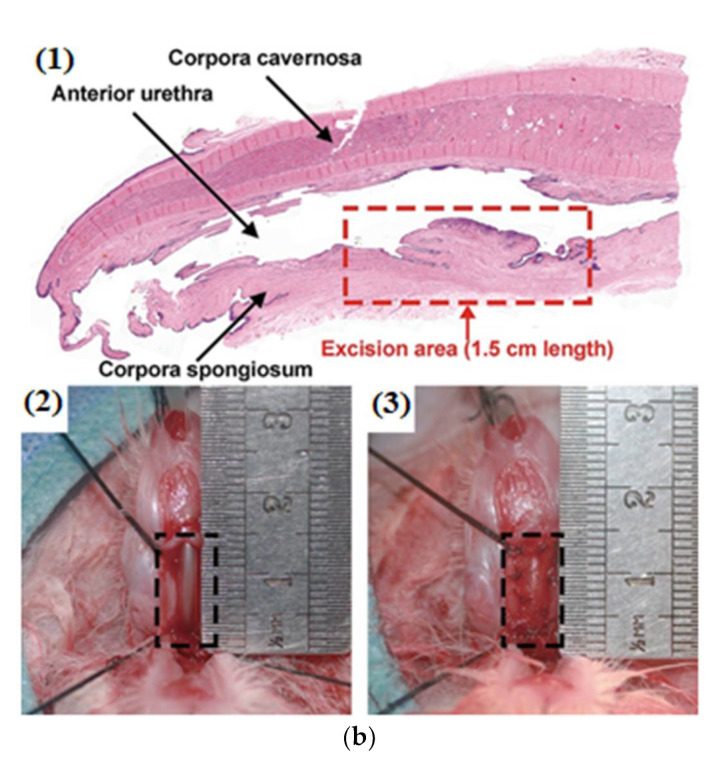
(**a**) Cellularized tubular autologous scaffolds and scaffold transplantation procedure. Scanning electron micrographs and fluorescent micro photos of the cross-section of each cellularized tubular autologous scaffold seven days after seeding (1). Left panels, scale bar 1 mm. Right panels, stained for keratin (AE1/AE3, green), elastin (red) in ECs, and actin (α-SMA, green), elastin (red) in SMCs on the cross-section of a tubular nanofibrous scaffold. Scale bar 400 μm. The cellularized tubular autologous PLLA/gelatine nanofibrous scaffold (2.2 cm in length) was sutured to the urethral defect site by an end-to-end anastomosis procedure (2). (For interpretation of the references to color in this figure legend, the reader is referred to the web version of this article.) (reproduced from [91] with permission of Elsevier, 2020), and (**b**) Substitution urethroplasty in vivo (1) Histological diagram of urethral defect rabbit model with an average length of a 1.5-cm ventral excision area. (2) Animal rabbit of the urethral defect. (3) Urethral reconstruction using composite scaffolds (reproduced from [92] with permission of Wiley, 2020).

In order to overcome the challenges facing the fabrication of 3D vascular scaffolds and smooth muscle cells [SMCs], many methods have investigated mimicking natural vascular 3D architecture to guide SMC action [90]. In this direction, temperature responsive shape-memory scaffolds are constructed for SMCs’ seeding. The scaffolds consist of polylactide–glycolide–trimethylene carbonate (PLGATMC) as an outer layer to control the rolling from planar to small-diameter tubular shape and an inner aligned orientation layer of nanofibrous membrane of polylactide–glycolide/chitosan (PLGA/Chi) to organize cell attachment, proliferation, and morphology. The experimental results indicated that smooth muscle cells’ properties and functions are based on the PLGA/Chi ratios of membranes, and the scaffold with PLGA/Chi ratio 7:3 induced the most appropriate function to culture SMC behavior. In addition, the PLGA/Chi@PLGATMC scaffolds transformed into a temporary planar at 20 °C for culturing and adhesion of smooth muscle cells and thereafter self-rolled into a 3D tube at 37 °C [90]. As reported, there are many challenges facing the development of engineered scaffold grafts that increase bioactivities and can effectively and quickly reconstruct urethral defects and rebalance the epithelialization of the urinary tract and other smooth muscles [91]. Hence, flexible poly(l-lactic acid/gelatin tubular nanofiber scaffolds with hierarchical architecture were prepared using electrospinning to regulate the phenotypic expression of ECs and SMCs to reconstruct urethral tissue. The fabricated nanofiber scaffolds presented high hydrophilicity and improved the adhesion, oriented elongation, and proliferation of New Zealand rabbit autologous ECs and SMCs simultaneously. By comparing the fabricated nanofiber scaffolds with pure PLLA nanofiber scaffolds, the fabricated PLLA/gelatin nanofiber scaffolds upregulated the expression of keratin (AE1/AE3) in epithelial cells and actin (α-SMA) in smooth muscle cells as well as synthesis of elastin. Three months of in vivo scaffold replacement of New Zealand rabbit urethras provided that tubular cellularized poly(l-lactic acid)/gelatin nanofibers scaffolds kept urethral patency and smoothed oriented smooth muscle cells remodeling, lumen epithelialization, and angiogenesis [91], Figure 6a.

Also, a stromal cell-derived factor-1-alpha (SDF-1α)-aligned-(SF)/3D porous bladder cellular matrix graft (3D-BAMG) hybrid scaffold for long-section ventral urethral construction and repair in vivo was developed, as represented in Figure 6b. Aligned SF microfiber/3D-BAMG and nonaligned SF microfiber/3D-BAMG scaffolds were fabricated via electrospinning and wet approach. The hybrid scaffolds controlled stromal cell-derived factor-1 alpha release for over sixteen days in vitro. Stromal cell-derived factor-1-alpha-aligned- (SF) nanofiber supported reforming of urethral mucosa, submucosal smooth muscles, and microvasculature, enhanced cellular proliferation, and minimized collagen formation. Stromal cell-derived factor-1 alpha expression was improved in regeneration of urethra at three months post-surgery in SDF-1α-aligned-silk fibroin group [92].

Considering the complexities of the native artery wall structure and the challenges facing present treatment strategies, many works have been investigated to develop biomimetic tri-layers tissue-engineered vascular grafts [93]. A bio-inspired tri-layer tubular graft, employing biodegradable polymers. was investigated using multiple technologies to act as natural vascular architecture. The inner layer, composed of polycaprolactone (PCL) nanofiber, presents considerable tensile strength and improves endothelial cell adhesion and proliferation. The middle layer, made of polylactic-co-glycolide (PLGA) with a 3D porous structure, favors vascular smooth muscle cell (SMCs) breakthrough. Polyurethane (PU) was chosen to be the covering layer, for holding the entire tubular structure, and thermal crosslinking obtains interaction among the three layers. The in vivo experiments of subcutaneous seeding for 8 weeks indicate the biomimetic tri-layer vascular graft could keep intimal integrity, cell infiltration, collagen deposition, and scaffold biodegradation [93]. Also, a novel vascular graft in a rat model with small diameter and degradability properties was investigated [94]. Electrospun conduits composed of degradable thermoplastic polycarbonate urethane (dPCU) were examined in short and long-term follow-up and compared with expanded-polytetrafluoroethylene (e-PTFE) controls. Anti-inflammatories were upregulated in dPCU conduits and significantly increased with time in vitro. DPCU and e-PTFE grafts possessed improved long and short-term patency rates (92.9% in both groups at 12 months) in the rat model without dilatation or aneurysm formation. Compared to e-PTFE, dPCU grafts provided transmural ingrowth of vascular specific cells causing a structured neo-vessel composition around the graft. The graft material diminished slowly, while the compliance of the neo-vessel increased over time [94].

On the other hand, nanofibrous polyurethane scaffolds immobilized with resveratrol drug were prepared and evaluated towards viability of endothelial and smooth muscle cells. The results indicated that the loading of resveratrol significantly enhanced tensile strength and Young’s modulus of the scaffold and they became closer to native vessels. The release profile resveratrol presented smooth release from the nanofibrous scaffolds. In addition, resveratrol-loaded nanofibers possessed increase in anti-thrombogenicity with respect to polyurethane alone, enlarging time of human blood clotting and decreasing hemolysis. Moreover, the resveratrol-immobilized nanofibers resulted in suitable antithrombotic function and composition of a monolayer on the scaffold surface of endothelial cells and decreased the growth of smooth muscle cells [95].

Generally, electrospinning technology succeeded in developing various types of nanofibrous scaffolds and creating tubular scaffolds which are utilized in vascular tissue engineering. Published works have investigated the performance of artificial vascular grafts over the last years and the considerable progress achieved in large-diameter ones. Many challenges still remain for small diameter vascular tissues, due to various reasons, such as thrombogenesis or intimal hyperplasia. Also, there are challenges to develop an engineered scaffold graft that increases bioactivities and can effectively and quickly reconstruct urethral defects and rebalance the epithelialization of the urinary tract and other smooth muscles [91]. Therefore, the fabrication of nanofibrous scaffolds with desirable characteristics is needed to mimic the structures and functions of the native extracellular matrix and to develop multi-layer scaffolds to mimic the tissues while ensuring the substrates have mechanical strength in the range of targeted tissues.

#### 3.1.4. Skin Tissue Engineering

Skin is the outermost and the most important barrier organ in the body and plays a vital role in protection of internal tissues from external damage [96,97,98]. Therefore, the protection of this barrier against damage is vital to prevent microorganisms from penetrating and forming infections in wounds and other dangerous side effects. Also, the protection and healing of wounds are very urgent factors in keeping patients safe and healthy [97,98]. Recently, the development of novel nanomaterials for wound dressing and antibiotic agents is one of the more important challenges facing current medical technological innovations [99]. Thus, many researchers have been motivated to fabricate an ideal wound dressing and antibiotics with appropriate properties using nanomaterials, such as different types of fabricated electrospun nanofibers to enhance wound healing. Electrospun nanofibers have opened new ways to synthesizing and fabricating of novel materials to be used in skin tissue engineering through nano/microscale polymeric fibers, inorganic/organic compositions, biomaterials, elastomers, and other types of materials.

Polymeric nanofibers can be incorporated with nanofillers, such as nanoparticles and nanotubes, to improve their superior properties and enhance applications in skin tissue engineering. Electrospun nanofibers of glucose (G)-reduced graphene oxide (rGO) (0–1.0 wt%) blended with PVA to form PVA/GrGO scaffolds, referred as (PG) scaffolds, and crosslinked chemically with acidic glutaraldehyde (GA) in acetone medium to mimic the (ECM) to apply in skin tissue engineering. Further increase in concentrations of G-rGO in PG scaffolds cause a decrease in tensile strengths and elongations, increasing the thermal properties. The biological properties of PG scaffolds were examined using in vitro hemolysis, using CCD-986Sk (a human skin fibroblast cell line). Results indicated G-rGO incorporation in PVA nanofibers induced a small shift from hydrophilic to hydrophobic. Moreover, the PVA/G-rGO scaffolds did not possess hemolysis of red blood cells, even at a G-rGO immobilizing of 1.0 wt%, and PG-1.0 scaffold (with a GRGO loading of 1.0 wt%) there was good compatibility with fibroblasts and highly increased metabolic activity after seed for twenty-one days, as compared with PG-0 controls [96].

Another nanofiber scaffold was synthesized from PU and cellulose acetate via electrospinning. RGO/Ag nanocomposite and/or curcumin was incorporated with the materials attributed to the antibacterial activity of rGO/Ag-nanocomposite. The scaffolds could prevent both of Gram-negative and Gram-positive bacteria via direct contact with them. In vivo histopathological experiments demonstrated that the scaffolds of the blended rGO/Ag nanocomposites and curcumin showed excellent wound healing and could promote the healing rate of artificial wounds, which provides considerable biomedical potential for nanomaterials in wound healing [97]. Development of an optimized zein/GO nanocomposite nanofiber was investigated. Tetracycline hydrochloride (TCH) was mixed by immobilizing on GO nanosheets. The SEM photograph illustrated that the diameter of the nanofiber reduced with increased GO ratio. Moreover, the mechanical behavior of the zein nanofibers was significantly affected by blending up to 1.0 wt% of GO. Drug immobilized zein/GO nanofibers released over a long period compared to zein nanofibers. Moreover, cellular experiments provided that the content (GO) ratio up to 1.0 wt% improved adhesion and proliferation of cells [98].

**Figure 7:**
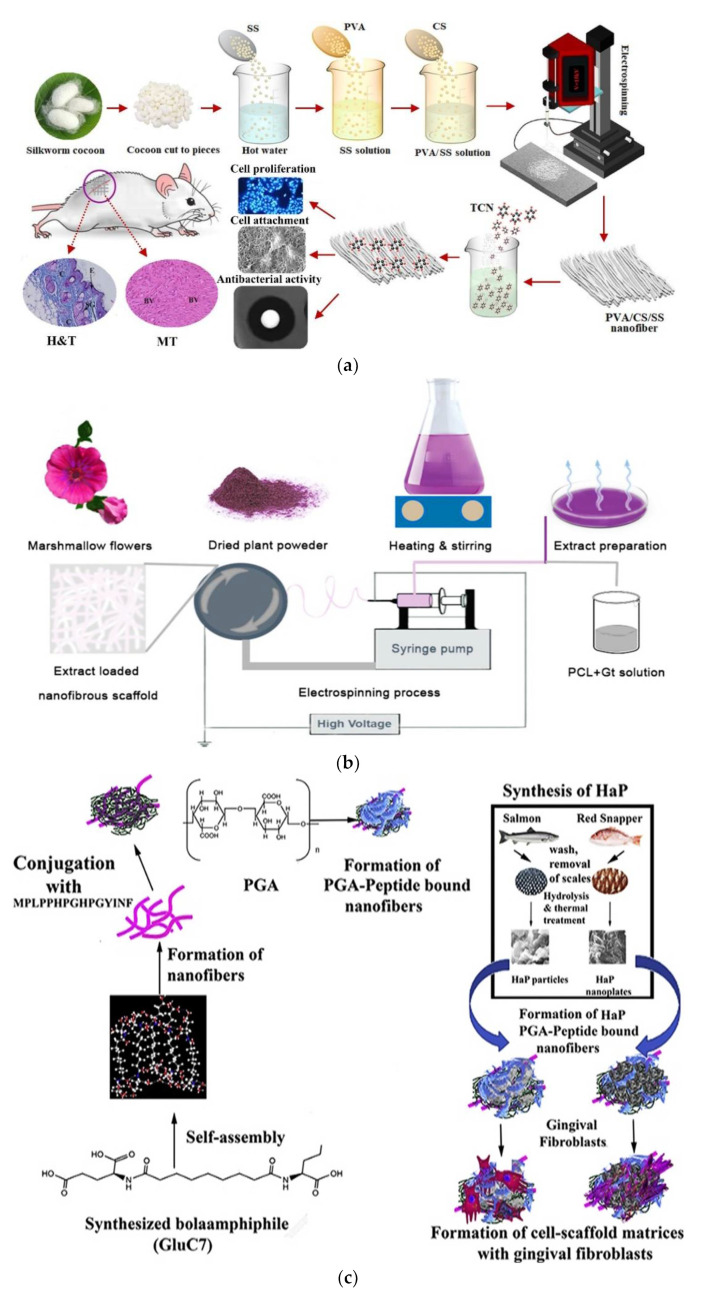
A schematic illustrates the fabrication of (**a**) PVA/CS/SS-TCN electrospun nanofibers (reproduced from [99] with permission of Elsevier, 2020), (**b**) electrospinning process for fabrication of AO extract loaded nanofibrous scaffolds (reproduced from [100] with permission of Wiley, 2019), and (**c**) the synthesis and development of the scaffolds and their interactions with gingival fibroblastsb (reproduced from [101] with permission of Elsevier, 2019).

On the other hand, many works illustrate nanofiber materials containing sericin being utilized to reconstruct epidermal–dermal tissues [99]. Therefore, PVA/Chi/SS- tetracycline (TC) nanofibers were synthesized using electrospinning. Polyvinyl alcohol (PVA)/chitosan Chi/silk sericin (SS)/tetracycline (TC) porous nanofibers, with size range from 305 to 425 nm, both in vitro and in vivo were investigated to apply to wound dressings. The prepared nanofiber presented excellent swelling properties, the addition of sericine caused increased hydrophilicity and elongation, while reducing the diameter of the fiber and its mechanical strength. Furthermore, fibroblasts (L929) seeded on the nanofibers with low sericin percent (PVA/Chi/1–2SS) showed high proliferation compared to those on sericin free nanofibers (PVA/Chi). Nanofibers incorporated with high sericin and TC amount highly prevent the growth of *Escherichia coli* and *Staphylococcus aureus*. A schematic illustrates the fabrication of PVA/Chi/SS-TC electrospun nanofibers was represented in Figure 7a. In vivo studies relating to PVA/Chi/2SS-TC nanofibers indicate improved wound healing, re-epithelialization, and collagen deposition compared with conventional gauze and nanofiber free sericin [99]. One of the most important extracts is the Althea Officinalis (AO) which was utilized to heal skin damage for a long time due to its promising properties. AO extract with various concentrations of (0, 5, 10, 15, and 20 wt%) were inserted into the nanofibrous scaffolds to test their ability for skin tissue repairing. The nanofiber scaffolds were fabricated through electrospinning by a mix of poly ε-caprolactone with gelatin. It was found that 15% of AO is considered the optimal amount introduced in the scaffold for excellent acceleration of skin tissue repair [100]. The electrospinning processes to fabricate AO extract-loaded nanofibrous scaffolds is represented in Figure 7b. In 2020, Niyasha et al. [101] investigated, for the first time, scaffolds functionalized with a segment of tyrosine-rich amylogenin protein peptidem followed by polygalacturnonic acid and hydroxyapatite derived from fish scales.

New peptide-based composite scaffold was fabricated by incorporating two varied fish scale-derived hydroxyapatite with modified peptide nanofibers to apply in periodontal tissue regeneration. The nanofibers were synthesized by self-immobilization of the newly constructed peptide bolaamphiphile Bis (*N*-α-amido-glutamic acid) 1,7 heptane tetracarboxylate and functionalized with a mix of the tyrosine-rich amylogenin peptide sequence MPLPPHPGHPGYINF followed by polygalacturnonic acid and HP derived from salmon or red-snapper fish scales. The scaffolds-based salmon scale derived HP exhibit larger mechanical strength and Young’s Modulus than snapper scale-derived scaffolds, as shown in Figure 7c. The bioactivity results indicated snapper scale-derived hydroxyapatite showed high alignment of the cells and greater differentiation ability into osteoblast-like cells [101].

A novel nanofiber scaffold was designed for the tissue remodel of periodontal bone, which consisted of electrospun PLA and calcium alginate (CA) nanofiber scaffold. The fabricated nanofibers, PLA/CA, exhibit a homogenous surface morphology with elongation at break more than pure PLA). The addition of CA increases the hydrophilicity of polylactic acid nanofibers, as well as supporting cell attachment of periodontal ligament cells (PDLCs) and bone marrow stromal cells (BMSCs). Moreover, the calcium alginate enhances the cell mineralization gene’s expression level and the composition of mineralization knots (BMSCs). On the other hand, both neat polylactic acid nanofibers and polylactic acid/calcium alginate nanofibers could enhance the expression level of inflammatory mediators and (TLR4) of human periodontal ligament cells (hPDLCs) [102].

Inorganic/organic compositions incorporated into porous biomaterials could be convenient for skin tissue engineering. Therefore, much research has been done to study the suitability of carbon nanofibers (CNFs) in utilization as a reinforcement agent in the enhancement of bone scaffolds [103]. Therefore, CNFs were functionalized using a 3:1 *v*/*v* mixture of concentrated sulfuric (H_2_SO_4_) and nitric acid (HNO_3_) for various periods. The characterization results exhibit the presence of –COOH and –OH groups due to the oxidation process. Furthermore, a composite polycaprolactone (PCL), as a matrix, and the CNF, as the reinforcement, was fabricated. The storage module of the acid treated CNFs was enhanced compared with untreated CNFs. Furthermore, the scaffolds of PCL/oxidized CNFs improve the cell proliferation [103].

In some particular wounds, such as nonhealing diabetic ulcers, bioactive glass (BG) materials have been recognized to bond well with hard and soft tissues. The reactions of such bioactive materials with biological fluids can enhance the vascularization of endothelial cells (ECs) and increase osteogenesis and angiogenesis [104]. In this regard, a silver-compositing bioactive glass by sol-gel approach is incorporated with the antibacterial bioactive glass nanofibers (ABGnf, dia 200–900 nm) using an electrospinning route, andwas examined both for wound-healing potential and antibacterial efficacy. An in vitro cell proliferation/migration analysis was investigated using SV-transformed GM00637 (skin fibroblast) cell line. The experimental results demonstrated that ABGnf possesses high cell proliferation (82%) compared to the control (47%) and ABGnf without boron (65%) within 24 h. The evaluation of antibacterial activity using *Staphylococcus aureus* was carried out in vitro and explored the inhibition zone to be two folds of the control group treated with a model aminoglycoside antibiotic, tobramycin [104].

Hydroxyapatite (HA) is an important biomaterial and has unique properties in versatile biomedical applications such as skin tissue engineering and recently in wound healing. Therefore, many works have studied the antibacterial behavior of HA structure and the influence of silver (Ag) ionic dopant into HA structure and their antibacterial behaviors [105]. Therefore, AgNPs were precipitated at interval times of exposure on the surface of polycaprolactone (PCL) electrospun nanofibers holding carbonated hydroxyapatite (CHA), which is partially replaced by selenite ions. The pulsed laser deposition approach formed this precipitation. The yield composite was developed towards adhering to the human osteoblast cell line (HFB4) after three days of in vitro culture. HFB4 cells lines were shown to proliferate and spread via the nanofibrous compositions. Moreover, the antibacterial property achieved about 45.0% at t = 20.0 min for *E. coli* compared with the ciprofloxacin activity, while it monitored 43.0% for *S. aureus*. Furthermore, increasing the deposition time of nanoparticles for AgNPs@Se-CHA/PCL improves both cell growth and bacterial inhibition [105].

Recently, various types of biomaterials have been fabricated and investigated to apply in skin tissue engineering applications, especially for serious skin defects. Many works investigate nanofibrous scaffolds of chitosan (Chi), and its compounds as a wound dressing, due to its multiple characterizations. Therefore, further modifications for chitosan nanofibrous scaffolds have been carried out to enhance its broad-spectrum antimicrobial activity [106,107,108]. A series of antibacterial, anti-oxidant, and electroactive nanofibrous membranes were prepared by electrospinning polyε-caprolactone (PCL) and quaternized chitosan-graft-polyaniline (QChiP) polymer solutions which collect the better mechanical property of PCL and multi-functionality of QChiP. The nanofibrous wound dressings possess electroactivity, the same mechanical manners as soft tissue, free radical suppressing ability, antibacterial behavior, and biocompatibility. In particular, PCL/QChiP15 (15 wt% of QChiP in the material) exhibited a developed balanced capability among antibacterial activity and cell proliferation, which more highly increased the wound healing process in a mouse full-thickness wounds defect model than a commercial dressing (Tegaderm™ Film) and pure PCL (PCL/QChiP0) nanofibrous membrane. Moreover, the results indicated that the wounds cured by PCL/QChiP15 nanofiber dressing presented high collagen deposition, granulation tissue thickness, and more angiogenesis [106].

Due to the unique surface properties of graphene oxide (GO), it is widely used as a new antimicrobial agent to prevent bacterial infections [107]. Graphene oxide nanosheets were anchored on shell Chi-core (L-polylactic acid, PLLA) designed nanofiber scaffolds to generate a synergistic microenvironment for wound healing. The SEM and AFM investigations indicated that GO donated Chi/PLLA nanofibrous wrinkles and rougher structure than those related to Chi/PLLA nanofibers. Furthermore, graphene oxide coating the nanosheets highly enhanced the hydrophilicity of Chi/PLLA nanofibrous scaffolds and improved its antimicrobial ability towards Gram-negative *Escherichia coli* (*E. coli*) and Gram-positive *Staphylococcus aureus* (*S. aureus*). Meanwhile, they support the proliferation of pig iliac endothelial cells (PIECs). Rats’ wounds treated by GO-coated Chi/PLLA nanofibrous scaffolds were more excellently cured than other groups on pathological section [107]. Furthermore, electrospun multilayer nanofibrous patches with a new structure were prepared using PCL and chamomile assembled carboxyethyl chitosan (CEChi) and PVA, in which chamomile extract was utilized as an antioxidant/antibacterial agent. Multilayer patches consisted of hydrophilic chamomile incorporated CEChi/PVA nanofibrous layer, which would contact the wound, and a hydrophobic PCL nanofibrous layer to reinforce the electrospun material. The SEM images demonstrated continuous, smooth, and bead-free nanofibers with smooth compatibility among polymers and chamomile. The mats showed appropriate tensile strength and antioxidant properties. Also, 15–30 wt% chamomile incorporated materials have excellent antibacterial ability, which improved with further increase of the content of chamomile. The findings showed that the Fickian-Diffusion mechanism controls chamomile release. The MTT analysis provided excellent cell viability for all materials except those with 30 wt% chamomile contents [108]. Also, developing an ideal wound dressing material was investigated, where scaffolds from gum tragacanth (GT), polycaprolactone (PCL), and poly vinyl alcohol (PVA) were prepared by electrospinning technique for applying to the healing of diabetic ulcers. The experimental results presented that the PCL-GT-PVA with a ratio of 2:2.2:0.8 and more GT in its build and average diameter 130 ± 19 nm was the desired composition. Mesenchymal stem cells on the scaffolds provided adhesion and proliferation of cells. Histological assessment of membranes, including stem cells in rats with diabetic ulcers, presented tissue repair and regeneration involving reepithelization and collagen deposition after 15 days [109]. Since many nanofibrous materials can act as extracellular matrix (ECM) protein fibers of skin tissues, Chi-alginate (Chi-Alg) nanofiber healing with different contents of gentamicin (Gn; 0–10 wt%) were prepared via electrospinning and treated as a drug delivery system. The Gn-incorporated nanofibers possessed excellent inhibition of bacterial growth, and the antibacterial activity enhanced with increased Gn content in the nanofiber. In vitro cell seeding tests showed Chi-Alg wound healings with 1–3% Gn induced L929 cell adhesion and proliferation more than materials with different Gn concentrations. In vivo studies indicated that Cs-Alg nanofibers contained 3% Gn highly improved skin-repairing in a Balb/C mice model by stimulating the composition of a thicker dermis, enhancing collagen formation, and developing the composition of new blood vessels and hair follicles [110].

**Figure 8:**
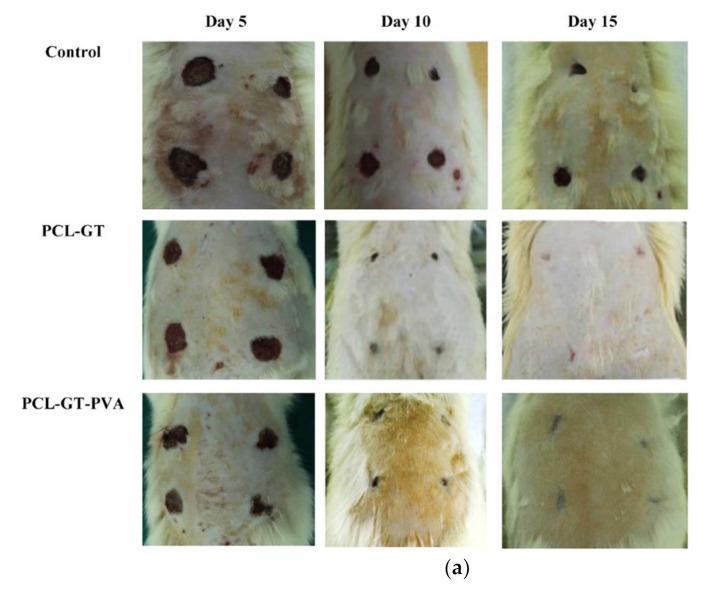

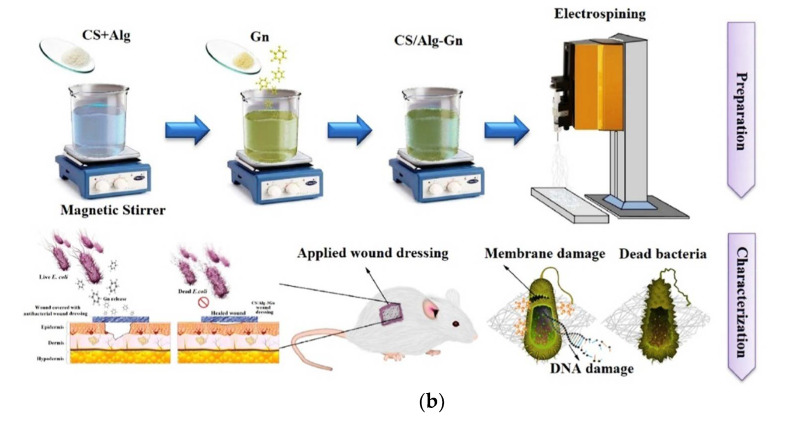
A schematic illustrates the fabrication of (**a**) Wound closure for rats after 5, 10, 15 days (two above wounds treated with acellular scaffolds and two lower wounds treated with cellular nanofibers (reproduced from [109] with permission of Elsevier, 2020), and (**b**) CS/Alg-Gn electrospun nanofibers (reproduced from [110] with permission of Elsevier, 2019).

Also, electrospun curcumin (Cur)-mixed Chi/PVA/carbopol/PCL nanofibrous composite for concurrent delivery of the buccal fat pad-derived mesenchymal stem cells (BFP-MSCs) and Cur to a full-thickness wound on the mouse model. Results showed that the prepared composite scaffolds have an affinity for cell culturing and promote their growth and proliferation, as shown in Figure 8a. Macroscopic and histopathological characteristics were measured at the end of seven and fourteen days after surgery, and their findings indicated that the prepared scaffolds groups reduced the period required for the wound healing process concerning the control group. Among those, scaffold/Cur, scaffold/Cur/BFP-MSC, and scaffold/BFP-MSC groups donated more efficient wound healing [111]. A schematic illustrates the fabrication of Chi/Alg-Gn electrospun nanofibers.

A biomimetic small intestinal submucosa (SIS) -based biocomposite (Chi/ES-SIS) for abdominal wall healing, chitosan (Chi), and elastin (ES) electrospun nanofibers were utilized to develop biodegradability, antibacterial activity, and angiogenesis. The results showed that the dialysis rate of Chi/ES-SIS composites was lower by about 24.5% than that of SIS. Moreover, the Chi/ES-SIS composites exhibited high microstructure stability and micromechanical characteristics compared with SIS. The antibacterial activity of Chi/ES-SIS composites towards *E. coli* and *S. aureus* were 98.87% and 98.26%, respectively, while the SIS provided no antibacterial ability. Finally, in vivo experiments presented that the Chi/ES-SIS composite could support tissue repair upon implantation without any inflammatory reaction [112].

Gelatin is a hydrophilic, adhesive, and biocompatible polymer widely utilized in wound healing systems and as an absorbent pad during surgery, where it is a bio-protein that contains high amounts of proline, glycine, and hydroxyproline [113,114,115]. Therefore, blending some materials with such polymers can enhance the desired characteristics in a vast gamut of medical applications [113,114,115]. So, during the last decade many works have fabricated novel nanofibrous scaffolds containing gelatin. Electrospinning of Gelatin (Gel) and Poly-3-hydroxybutyric acid (P), blended with anionic drug AgSD immobilized hydrotalcite (L) (L-AgSD) to prepare nanofibrous scaffolds which would interact with the native extracellular matrix appropriately for cutaneous regeneration. Antimicrobial tests confirm the possible activity versus microbial infection. Moreover, 86% of the loaded drug release of in seventy-two hours. In vitro biocompatibility experiments employing the NIH 3T3 fibroblast cell line presented high adhesion and proliferation of the cell conforming to the biocompatible property of the scaffold. The matrix speeds up healing of Pseudomonas-infected burn wounds on rat models [113].

Also, cinnamon (cin) was immobilized into poly(ε-caprolactone)/gelatin (PCL/Gel) nanofibrous matrices to produce a suitable material to enhance wound healing. Fabricated materials with cinnamon and free of cinnamon were employed to cure full-thickness excisional wounds in Wistar rats. The findings provided that the cinnamon content directly induced porosity, mechanical features, swelling, water contact angle, rate of transmission of water vapor and cell proliferation. Moreover, the data obtained from the in vivo test demonstrated that wound closure was (98%) after 14 days curing with PCL/Gel 5%, which was the best among other groups [114]. Another hybrid blend of nanofibrous membranes consisting of poly l-lactide-co-d,l-lactide (PLDLLA) and PVA, including triclosan (Tri), as antibacterial drugs were developed. The results showed that the hybrid material exhibited improved hydrophilicity with water contact angle (WCA) of 53° over the blend material with WCA of 73° which was further confirmed by a swelling test. The antibacterial performance presented excellent results for hybrid-Tri with inhibition zones of 35 mm and 48 mm for *E. coli* and *S. aureus*, respectively. Also, the hybrid membrane exhibited significant mechanical behavior. Furthermore, the SNL 76/7 fibroblast cell line culture indicated that the hybrid-Tri nanofibrous sample possessed more excellent proliferation performance than the blend-Tri sample attributed to minimal cytotoxicity and maximal cell viability by MTT and acridine orange/ethidium bromide staining [115].

On the other hand, fabricated electrospun nanofibers that contain gelatin were investigated in wound healing applications as composite scaffolds, and as efficient artificial biomimetic skin substitute nanofiber membranes in vitro and in vivo studies [116,117,118]. Polycaprolactone–gelatin (PCL–gel) hybrid nanofibers were prepared by two nozzle electrospinning. Cellulose nanocrystals (CNC) were added into the gel and PCL nanofibers to modify their features, where the presence of CNC leads to an increase by 80% and 60% in modulus tensile strength. In vitro experiments revealed that CNC incorporation increases scaffold degradation rate by 25%. However, the MTT analysis, cell morphology, and fluorescence staining tests exhibit that CNC does not affect the nanofiber biocompatibility, and cells could grow, differentiate and coat the scaffold surface. Scaffolds with and without CNC supported powerful wound healing in Balb/c mice [116].

Moreover, biomimetic skin substitute properties of PLLA/Gel nanofiber membranes were evaluated in facilitating chronic cutaneous wound healing. The material physical and biological characteristics were evaluated. Clinical applications further confirmed safety and effectiveness. The proliferation of fibroblasts and monocytes was highly enhanced at the seventh day seeded on the substitute with obviously inhibited bacterial growth. In the clinical case experiment, the wound healing closed with 93.3%, and the recovery rate was 6.7% [117]. Also, bacterial cellulose nano-crystal (BCNC) involving PCL/Gel nanofibrous composite scaffolds were prepared using electrospinning for simulating the extracellular matrix of GBM tumor. The diameters of the fiber in the matrix were increased with the increased concentration of BCNC. Further increase in BCNC led to a change in fiber morphology from smooth to beaded form. In-vitro nanofibrous scaffolds bioactivity was evaluated with U251 MG glioblastoma cells and developed cell adhesion and proliferation in the case of PCL/Gel/BCNC compared with PCL/Gel. Moreover, PCL/Gel/BCNC was favored to improve axon growth and elongation, supporting communication among tumor cells and the microenvironment, inducing tumor recurrence process [118].

Bio-based PLA polymers are becoming the most important biomedical polymers due to their promising properties, such as considerable cytocompatibility, biodegradability, processability, and mechanical characteristics [119]. Thus, the morphology and bioactivity of electrospun polylactic acid/siliceous sponge spicule (SSS) nanofibers were examined. The tensile, thermal, and water-resistant properties of the fibers were also evaluated. The results exhibited improvements in polylactic acid’s thermal and tensile features, increasing the siliceous sponge spicules ratio (specifically, a 3 wt%). Moreover, there was increase in the contact angle of PLA/SSS with increasing siliceous sponge spicules content, compared with polylactic acid nanofibers. Finally, PLA/SSS nanofibers presented softly improved human foreskin fibroblast cell proliferation, excellent cytocompatibility, and antibacterial activity [119]. Also, poly mannitol sebacate/poly lactic acid (PMS: PLA) nanofibrous scaffolds were fabricated using the electrospinning method. At 10% *w*/*v* for PMS: PLA 60:40A homogenous nanofibers of size 235 ± 38 nm resulted. The in vitro and in vivo biocompatibility tests provide high cytocompatibility, proliferation, and tissue responses of PMS: PLA nanofibrous scaffolds with suitable interactions between cell and scaffold. Moreover, inflammatory responses of PMS: PLA nanofibrous scaffolds were negligible [120].

Clinical research illustrated that keratin can stimulate keratinocyte cells existing in the wounds and activate these cells to quickly enter a hyper-proliferative phase [121]. Therefore, varied hydroxyapatite (HA) contents loaded by sodium hexametaphosphate were mixed with a keratin/polyethylene oxide (PEO) spinning system to prepare reinforced keratin blend nanofiber nonwoven membranes as a potential candidate for wound healing. The tensile strength of keratin incorporated nanofiber membranes with the presence of modified HA ratio 15% was 2-fold more than that of the HA free. The findings indicated that the Keratin/PEO/HA nanofiber mesh significantly enhanced L929 cell proliferation, minimizing inflammatory response in the infective area and improving the skin remodeling process in the subsequent curing stages [121].

Recently, elastomers have been extensively used to investigate cell physiology in fields such as mechanobiology, but their undesirable properties, such as intrinsic high hydrophobicity, render its surface discordant with prolonged cell adhesion and proliferation. So, many works have been done to improve the mechanical and chemical stability bonding between an elastomeric bulk substrate and electrospun nanofibers, limiting the presence of fibers to a thin interfacial layer [122]. To achieve this, elastomers composed of vulcanizing silicone (RTV), polydimethylsiloxane (PDMS) as well as functionalized PDMS-based materials were selected as wafer substrate for the capture of polyvinylidene fluoride-co-hexafluoropropylene (PVDFhfp) fibers, as antithrombotic polymer. Electrospun fibers onto modified interfaces do as conjugated agents on wafers, supporting breakthrough and composition of a stable link among the fibers surfaces and the elastomers after treating the interface. Cultivating dermal fibroblasts using these conjugated membranes enhanced cell attachment and growth over 7 days with respect to the composition of the substrate, providing suitable cytocompatibility for all composite materials [122].

During the last decade, many biomaterials have attracted great attention. One of these promising biomaterials is silk fibroin (SF) which is a natural protein, has abundant supply, unique biological characteristics and can be reformed into various structures [123]. Biomimetic (SF)/(PCL) matrices were prepared using co-electrospinning, followed by deposition of positively charged (Chi) and negatively charged type I collagen (COL) on the nanofibrous metrics via electrostatic layer-by-layer (LBL) self-immobilized approach. As a result, the average diameter of SF/PCL increased accordingly through the LBL process which confirmed SEM photographs. In addition, the LBL coated materials improved mechanical function and hydrophilicity of the materials. Moreover, LBL deposited materials enhance antibacterial activity and support cell adhesion, growth and proliferation. Ultimately, in vivo wound dressing analysis in rat models presented that layer-by-layer covered materials accelerate the wound closure time, improve collagen deposition and relieve extra scar formation via TGF-b/Smad signaling pathways, which provided the support application of the nanofibrous materials in skin remodeling [123]. Also, a novel type of chimeric spider silk proteins (spidroins) NTW1–4CT was mixed with polyl-lacticco-ε-caprolactone (PLCL) to form nanofibrous scaffolds via electrospinning. Spidroins consisted of an N-terminal module (NT) from major ampullate spidroins, a C-terminal module (CT) from minor ampullate spidroins and 1-4 repeat modules (W) from aciniform spidroins. The increase in W modules content will lead to increase in both the tensile strength and elongation of blend scaffolds. In vitro experiments, using human umbilical vein endothelial cells (HUVEC) seeded on NTW4CT/PLCL (25/75) scaffolds possessed higher activity of proliferation and adhesion than on pure PLCL scaffolds [124].

Another vital adhesive protein that has attracted considerable attention and been utilized as material surface modification is 3,4-dihydroxyphenylalanine (DOPA) and its derivatives which are extracted from marine mussels [124]. DOPA was used as basic fibroblast growth factor (bFGF) and ponericin G1 (PonG1), using tyrosine hydroxylation, which are considered to have high attachment ability to material surfaces. DOPA-bFGF and DOPA-PonG1 were employed for surface functionalization of polylactic-coglycolic acid (PLGA) electrospun nanofibrous scaffold for skin wound repair. The findings regarding the DOPA-bFGF- and DOPA-PonG1-loaded PLGA nanofibrous films showed bionic activity, enhanced tensile strength, and hydrophilicity. DOPA-bFGF and DOPA-PonG1 presented stronger conjugation affinity to PLGA films compared with bFGF and PonG1. Moreover, DOPA-bFGF and DOPA-PonG1 films can highly support BALB/c 3T3 cell adhesion, proliferation and tissue regeneration related gene expression. In vivo studies showed that DOPA-PonG1/DOPA-bFGF@PLGA nanofibrous films reduce wound healing time, speed up epithelialization and corroborated skin remodeling [125]. Due to the fascinating biological properties and biomimetic structures of electrospinning of pure alginate and its derivatives, it has gained considerable attention in biological fields [126]. So, alginate dialdehyde (ADA) with developed and adjustable chain flexibility was synthesized by periodate-oxidation. The corsslinked nanofiber membranes ADA that resulted showed better mechanical features and adjustable degradability. Moreover, biocompatibility studies provided that the fabricated membranes were noncytotoxic and could support cell adhesion and proliferation [126].

To circumvent many limitations facing the applications of some electrospun nanofibers, such as poor mechanical properties, an online suture approach to prepare electrospun polycaprolactone (PCL) fibrous yarns incubated with both collagen (COL) and bFGF growth factor, to prepare bFGF-COL@PCL sutures was devised. The in vivo experiments revealed that, as compared to commercialized vicryl suture, bFGFCOL@ PCL sutures highly support wound healing at various stages by decreasing granulation tissue building time, collagen formation, and re-epithelialization. The improved wound healing activity of bFGF-COL@PCL sutures is referred to two synergistic factors: (i) the excellent-oriented nanofibrous texture minimizes tissue hindrance to reduce their trauma and (ii) the introduction of both collagen and bFGF improve the basement membrane (BM) reconstruction, cell proliferation, and angiogenesis [127]. Also, electrospun membranes were prepared by mixing gelatin and PEG methacrylate (GelMet) with a concentration ratio (14 wt%, 17 wt%, and 20 wt% GelMet). Keratinocytes, hair follicle bulge stem cells (HFBSCs), and fibroblasts were successfully separated and seeded in 14 wt%, 17 wt% and 20 wt% GelMet scaffolds and created a tri-layered electrospun structure. Due to plasticity, by adding HFBSCs, the cell content of substitute skin was expected to increase without further addition of various cell populations. The diameter of the fiber and pore size of the scaffold in the various layers were synthesized to simulate the original structural of the collagen matrix through the native skin [128].

Recently, due to the unique physicochemical properties of graphene and its derivatives, it has been widely used as an additive in the field of biomaterials to improve mechanical performance, hydrophilicity, and interaction between cell and scaffold [129]. Highly flexible nanocomposite nanofibrous scaffolds composed of polycarbonate diol and isosorbide-based (PU) and hydrophilic (GO)NPs are incorporated at ratios up to 8%. The addition of nano-GO improved the hydrophilicity, elasticity, and stress relaxation capacity of the polyurethane-derived nanofibrous scaffolds. When seeded with C2C12 cells, the polyurethane–nano-graphene oxide (PU-GO) nanofibers increase cells’ initial attachment and spreading and further the proliferation. Furthermore, the PU-GO scaffolds highly up-regulated the myogenic mRNA levels and myosin heavy chain expression. By applying dynamic force, the cells showed higher myogenic differentiation markers at both gene and protein levels and presented more aligned myotubular structure [129].

Due to the versatile properties of (PVA), such as its nontoxicity, water-soluble synthetic-polymers, considerable biocompatibility and eventual biodegradability in the body, it has great attention as a hydrophilic polymer in biomedical applications [130,131,132]. The highly hydrophilic PVA was transformed into water-insoluble electrospun fibers utilizing citric acid (CA) as a green crosslinker. The crosslinked nanofibers showed higher water stability than un-crosslinked fibers even after 72 h soaking in water. Moreover, crosslinked PVA nanofiber enhances strength, elongation, and thermal stability properties. Also, crosslinked PVA nanofibers possess high stability in cell culture media for up to 96 h and promote NIH3T3 mouse fibroblast cells [130]. Also, a biaxial electrospinning strategy was utilized to prepare cellulose nanocrystal CNC blended PCL-PVA/NaAlg nanofibers. Afterward, sodium alginate was crosslinked via incubation of the material in a CaCl_2_ aqueous solution. Mechanical experiments indicated that the addition of CNC improved the tensile modulus by 65%.

Moreover, the crosslinked samples raised elongation at break and improved the sample wettability. In addition, Cell NIH/3T3 viability was highly enhanced (90%) with the addition of CNC to PCL-CaAlg nanofibers [131]. Another electrospun Santa Barbara Amorphous (SBA)-15-mixed (PVA) with curcumin was prepared and utilized as a biomimetic nanoscaffold for skin tissue engineering. Curcumin was chosen for its antimicrobial and anti-inflammatory performance, and SBA-15 acted as a drug carrier. The nanofibrous material was found to improve cell migration, proliferation, cytocompatibility, and biocompatibility without any cytotoxicity, which was confirmed from the findings of MTT assessment of cell attachment, and live/dead analysis utilizing HaCaT cells. The observations of the antibacterial activity indicated that the prepared nanofiber composed a potent material for skin wound-healing therapeutics. The in vitro drug release profile controlled over 80 h presented a smooth release pattern of curcumin from the SBA-15-encapsulated PVA nanofiber. The in vivo findings indicated that SBA-15-composited PVA nanofiber with curcumin presented performed wound-healing activities [132].

The mechanical characteristics of the scaffolds are critical factors. Much work has been done to develop alginate nanofibers. A two-step approach for coaxial electrospinning and post electrospinning is an efficient experiment for preparing superfine nanofibers with highly swellable hydrogels. Alginate and PCL were co-electrospun through fibrous meshes with a coaxial nozzle; alginate was encapsulated in PCL and then cross-linked in calcium chloride solution. Polyε-caprolactone shell was dissolved from the meshes by washing with organic-phase. The cross-linked hydro=nanofiber possesses high stiffness and Derjaguin–Müller–Toporov modulus. Moreover, NIH 3T3 cells seed on the material presented deeply infiltrated to the bottom of the mesh [133]. Also, and to overcome these disadvantages associated with (COL), polyamide (PA) has been used to improve the mechanical property of drug loaded collagen [134]. (COL) combined with *N*-acetylcysteine to compose of controlled release and chemically crosslinked COL/NAC (*N*-acetylcysteine) hybrid with PA nanofibers to improve the mechanical function of COL and prepare this multi-layered (PA-COL/NAC) scaffold. The results showed that the prepared scaffolds had an excellent porous structure and swelling properties. Moreover, the PA-COL/NAC scaffold could simply release NAC over fourteen days. After implantation of the cell, the PA-COL/NAC scaffold exhibited enhanced cell proliferation and migration over the other groups. In vivo, PA-COL/NAC scaffolds could support wound dressing better than all other groups [134]. Recently, the development of layered structures composed of nanofiber nanofibrous material have attracted considerable interest as a new nanomaterial to mimic skin tissues in wound healing approaches. A novel hybrid bilayer material consisting of zein-based composite film and nanofiber layers was put forward as a wound dressing material. The upper layer was based on *H. perforatum* oil blende zein film containing MMT and the bottom layer was composed of 3D electrospun zein/MMT nanofibers to stimulate wound dressing with controlled release of *H. perforatum* oil. Results indicated that the produced monolayer films exhibited suitable mechanical and gas barrier features and surface wettability for wound dressing. *H. perforatum* oil was released from the prepared membranes up to 48 h. Bilayer metrics presented antimicrobial behavior against *E. coli, S. aureus*, and *C. albicans* without any toxic effect on NIH3T3 mouse fibroblasts and HS2 keratinocyte cell lines. In vitro scratch analysis results demonstrated that *H. perforatum* oil had a wound healing activity by encouraging fibroblast migration. The proliferation tests presented an increase in fibroblast proliferation on *H. perforatum* oil assembled bilayer membranes [135]. Also, a dual mesh, composed of two nanofibrous layers, was manufactured: the first layer consisted of (PGS/PCL) to promote the dressing of the abdominal wall defect and the second one was made of a nondegradable, nonadhesive smooth polycarbonateurethane (PU) nanofiber, with appropriate non adhesion function of the viscera to the mesh. To make the double-layered structure, PGS/PCL fibers were electrospun directly onto the polycarbonateurethane film, this technique resulted in a final product with well-conjugated layers. The tensile experiment indicated that the dual mesh possessed elongation behavior of 7-folds more than individual counterparts, simulating native tissue features. In vitro assessment with human umbilical vein endothelial cells provided the double function of the meshes, in which the PU layer did not permit cell attachment, whereas the PGS/PCL layer enhanced cell adhesion and proliferation [136].

High levels of hydrogen peroxide (H_2_O_2_) released from Inflammation or infectious wounds may prevent wound healing processes. Also, such reactive oxygen-species (ROS) have a considerable pivotal effect in healing processes, cascading at multiple stages [137]. Therefore, a H_2_O_2_-responsive smart dressing, prepared by mixing Eu CPs into (PAN) nanofibers meshes, where europium (Eu^3+^) Coordination Polymers (Eu-CPs) showed properties to determine the level of H_2_O_2_ and allow wound healing. Therefore, this theranostic wound dressing can assay H_2_O_2_ level of the wound microenvironment via a facile visible color change. In addition, in vitro cytocompatibility tests showed that human umbilical vein endothelial cells (HUVEC) and fibroblast cells (L929) could well adhere and proliferate onto the PAN-Eu-CPs nanofibers materials. Moreover, in vivo animal experiments provided that PAN-Eu-CPs nanofibers materials could reduce wound healing period by allowing neovascularization performed by immunohistochemical analysis [137].

Recently, developments in the fabrication of multifunctional electrospun nanofiber nanomaterials have significantly contributed to the enhancement of wound healing scaffold nanomaterials with notable potential for skin regeneration in vitro as well as in vivo. On the other hand, one of the biggestt challenges facing the wound healing during the last years is development of a novel smart wound dressings which have the ability to interact with the wounds and effectively facilitate wound healing with consiedrable sensing characteristics and multiple smart properties using smart nanomaterials as well as stimuli-responsive and self-healing nanomaterials [138].

Finally, Table 1 summarizes some of the recent electrospun nanofibers nanomaterials investigated in this review used in regeneration of tissues and organs.

## 4. Conclusions and Outlook

In this review, we discussed recent progress and potential applications of electrospinning nonofibers in biomedical applications. Also, this review represents enormous promising works which have recently been done to develop and improve the utilization and application of electrospun nanofibers.

Nowadays, electrospun nanofibers with superior performance have illustrated considerable and promising results for biomedical applications. Their unique characteristics makes them convenient to apply in various fields. However, fabrication of nanofibers with distinct morphological and superior properties and considerable yield is still challenging. Such fabricated nanofibers can be functionalized through various techniques to widespread application in different strategic fields. Hence, considerable developments have been achieved even though there are still various challenges. Much effort has been put into improving desired performance of fabricated electrospun nanofiber and functionalized nanofiber application in specific areas. However, further work still needs to be done to obtain more promising results and eventual practical electrospun nanofibrous materials by advanced electrospinning techniques and scaling up the fabrication of nanofibers from the laboratory to commercial scales, especially for biomedical applications. The development of nanomaterials/biomaterials from biodegradable, biobased electrospun nanofibers is imperative to improve the applications of electrospun nanofibers in diverse fields, such as biomedical applications.

Also, electrospun nanofiber materials can play a vital role to overcome many challenges facing tissue engineering technologies in regeneration of tissues and organs where the scaffolds fabricated by electrospun nanofibers materials can mimic 3D with other physiological properties of tissues and organs in vitro to then transplant in vivo. Also, they are promising materials to regenerate injured nerve through tissue engineering technologies. Another serious challenge facing tissue engineering technologies to mimic and fabricate nerve grafts is losing the structural integrity, or biological functionality, of some natural polymer nanofibers used in a media simulating that in the human body. Therefore, further intensive research is required to explore and fabricate new materials to overcome such challenges. Using nanomaterials has many disadvantages, such as aggregation, uncontrollable release, costs, and potential cellular toxicity. Therefore, further research is needed to avoid such disadvantages.

On the other hand, current utilization of electrospun nanofibers in biomedical applications, such as bone cell proliferation, nerve regeneration, vascular tissue, and skin tissue engineering has been demonstrated. Many of these results were obtained for small rodent models which may be accompanied by restrictions regarding their immunological responses compared with humans. Therefore, these studies must be confirmed with suitable, and larger, animal models.

## Figures and Tables

**Table 1:** Some of the recent electrospun nanofibers nanomaterials investigated in this review used in regeneration of tissues and organs.

Application	Nanofiber	NF Diameter (nm)	Contact Angle	Tensile Strength (MPa)	Break Strain (%)	Ref
Bone	collagen/polycaprolactone (PCL) (70:30%)	305.5 ± 85.8	59.9 ± 2.5°			[58]
	Gel/PCL (70:30%)	473.9 ± 136.8	61.7 ± 2.6°			[58]
	(PU-Gel)/(N6-Gel)	161 ± 79	40 ± 2°	1.96 ± 0.09	76 ± 4	[59]
	GEL/PDLLA/RKKP (18:54:29, %wt)	1330 ± 490				[60]
	CS/PEO (50/50, 1% GA)	114 ± 18		9.47		[62]
	crosslinked-PCL/CS/metformin	462 ± 98.75	44.25 ± 1.37	4.3 ± 0.9	4.2 ± 0.8	[63]
	TPU/CNF-PDA	576	35.8 ± 4.1	14.9 ± 2.2	205.7 ± 8.3	[66]
	BC/CA	820 ± 10	43.4 ± 6.0°	0.81 ± 0.02		[67]
	PLCL/BSA	130 ± 30	141.7°			[70]
	PCL non-aligned	505–738		4.02 ± 0.88		[75]
	PCL aligned			9.90 ± 0.87		
	coaxial PGS/PCL non-aligned			5.06 ± 1.51		
	coaxial PGS/PCL aligned			11.78 ± 0.73		
	CNFs	96 ± 14.8				[76]
	PTH-Fc/PLCL/SF	671.62 ± 109.01	125.77 ± 3.00°	12.28 ± 2.13		[77]
	PLLA-COL	86.94 ± 35.72	78.97 ± 4.04°	5.13 ± 0.28		[81]
	MS-COL		81.87 ± 3.48°	4.74 ± 0.12		
	PPM	500	30–60°	15		[82]
	PU/ghee/propolis	576 ± 144.96	55 ± 1°	22.02	194.06	[83]
Vascular	PLCL/TSF	408 ± 101	56 ± 8°	7.383 ± 0.3	147.75 ± 22.6	[89]
	PLLA/gelatin (50:50)	361.0 ± 114.6	<50°	18.7		[91]
	aligned SF/3D-BAMG composite			2.7 ± 0.2	108.1 ± 5.7	[92]
	nonaligned SF/3D-BAMG composite			2.4 ± 0.1	111.2 ± 3.7	
	PCL fibers	368.25 ± 200.79		4.68 ± 1.64	166.50 ± 23.45	[93]
	PLGA scaffold	--		1.04 ± 0.46	71.99 ± 32.12	
	PU fibers	732.90 ± 219.70		10.08 ± 4.20	187.20 ± 46.24	
Skin tissue engineering	PG-0	92 ± 24		5.96 ± 0.966	78.3 ± 3.9 34.5 ± 4.2 18.5 ± 5.7 9.0 ± 2.5 8.1 ± 3.4 11.5	[96]
	PG-0.05	100 ± 27		5.53 ± 1.3		
	PG-0.1	108 ± 25		5.13 ± 1.5		
	PG-0.3	173 ± 66		4.3 ± 1.2		
	PG-0.5	388 ± 67		2.6 ± 0.8		
	PG-1.0	316 ± 219		2.58 ± 0.43		
	UC	678 ± 235	115.38^°^	1.6	169	[97]
	UC-GS	254 ± 79	64.95°	3.75	92	
	UC-C	327 ± 136	116.50°	1.13	45	
	UC-GSC	222 ± 44	61.38°	3.4	54	
	Z45	230	84.76 ± 1.58° 76.26 ± 2.02° 68.39 ± 1.30° 64.26 ± 1.27°	4.83 ± 0.38 7.47 ± 0.82 10.32 ± 1.07 6.34 ± 0.43	3.85 ± 0.24 3.10 ± 0.41 2.96 ± 0.28 1.41 ± 0.23	[98]
	Z45GO0.5%	191				
	Z45GO1.0%	162				
	Z45GO1.5%	137				
	Z45GO1.0%-TCH	159				
	PVA/CS/0 SS	425 ± 34	42 ± 3.1°			[99]
	PVA/CS/1 SS	397 ± 29	--			
	PVA/CS/2 SS	371 ± 27	--			
	PVA/CS/3 SS	328 ± 27	24 ± 2.0			
	PVA/CS/5 SS	305 ± 26	--			
	PGA0	175 ± 27		6.7 ± 0.8	37.4 ± 2.8	[100]
	PGA5	183 ± 28		7.8 ± 1.3	34.7 ± 2	
	PGA10	191 ± 31		8.9 ± 1.3	33.8 ± 1.5	
	PGA15	235 ± 38		12.6 ± 0.3	32.5 ± 3.1	
	PGA20	189 ± 65		5.8 ± 1.5	36 ± 10.5	
	PLA	780 ± 200	>150° <30°	0.25 ± 0.03	31.2 ± 4.7	[101]
	PLA/CA1	250 ± 90		1.36 ± 0.10	79.3 ± 7.6	
	PLA/CA2	250 ± 90		1.41 ± 0.34	85 ± 0.53	
	PLA/CA3	250 ± 90		3.13 ± 0.24	110.8 ± 10.9	
	PCL	91 ± 24 135 ± 33 169 ± 38 174 ± 38 175 ± 35	-- 81.7%:48.1 (for Fiber contain QChiP)			[106]
	PCL/QChiP0					
	PCL/QChiP5					
	PCL/QCSP10					
	PCL/QChiP15					
	PCL/QCSP20					
	CECS/PVA	93 ± 19	42 ± 2.1°	16 ± 2.0	38.0 ± 9.0	[108]
	CECS/PVA/5 wt% chamomile	115 ± 24	45.1 ± 2.4°	13.1 ± 3.0	64.2 ± 12.1	
	CECS/PVA/10 wt% chamomile	137 ± 26	45.2 ± 2.8°	12.1 ± 3.1	54.3 ± 17.0	
	CECS/PVA/15 wt% chamomile	149 ± 33	41.8 ± 1.0°	9.1 ± 1.01	48.5 ± 11.0	
	CECS/PVA/20 wt% chamomile	153 ± 34	44.7 ± 3.2°	8.9 ± 2.8	48.2 ± 6.01	
	CECS/PVA/30 wt% chamomile	183 ± 63	42.8 ± 2.6°	8.2 ± 1.0	49.1 ± 12.10	
	PCL/Gel	891 ± 64:984 ± 97	53.19 ± 4.06°:58.73 ± 1.16°	2.83 ± 0.8:2.23 ± 0.6		[114]
	PCL/Gel/1%cin					
	PCL/Gel/5%cin					
	PCL/Gel/25%cin					
	PLDLLA	300–450	129	20 ± 0.8	28 ± 1	[115]
	PLDLLA/PVA hybrid	275–425	53	19 ± 0.9	32 ± 1.5	
	PLDLLA/PVA blend	275–300	73	13 ± 0.5	48 ± 2.1	
	PLLA/Gel			4.42 ± 0.43	80.39°	[117]
	CL-PVA/NaAlg	170.1 ± 44	91 48°	10.0 ± 1.9	53 ± 8	[131]
	PCL-PVA/NaAlg+CNC	172.1 ± 90		7.4 ± 2.2	65 ± 9	
	PCL-CaAlg	171.0 ± 60		9.5 ± 2.5	67 ± 9	
	PCL-CaAlg+CNC	216.4 ± 89		10.1 ± 0.6	81 ± 1	
	PVA 8%	194 ± 76	52.4° ± 1.3			[132]
	PVA 10%	352 ± 118	52.4° ± 1.3			
	PVA+SBA-15 (1%)	175 ± 57	--			
	PVA+SBA-15 (3%)	325 ± 61	--			
	PVA+SBA-15 (5%)	216 ± 39	--			
	PVA+curcumin	181 ± 77	56.5° ± 0.7			
	PVA+SBA-15 (5%) + curcumin	234 ± 77	48.2° ± 1.4			

## Data Availability

Data is contained within the article.

## Nomenclature

1D	one dimension
2D	two dimensions
3D	three dimensions
ECM	extracellular matrix
ENFSs	electrospun nanofiber scaffolds
PmNFs	polymer nanofibers
BEPCs	bone marrow endothelial progenitor cells
PCL	polycaprolactone
Gel	gelatin
N6	nylon 6
PU	polyurethane
ALP	alkaline phosphatase
La	lanthanum
Ta	tantalum
GAG	glycosaminoglycan’s
Chi	chitosan
CH_3_COOH	acetic acid
PEO	polyethylene oxide
CM	carboxymethyl
hMSCs	human bone marrow-derived mesenchymal stem cells
C	cellulose
LA	lactic acid
TPU	thermoplastic polyurethane
CNF	cellulose nanofibrils
PDA	polydopamine
BC	Bacterial cellulose
PLA	poly(lactic acid)
RC	regenerated carbon
PANI	polyaniline
BSA	Bovine serum albumin
PDGF-BB	platelet-derived growth factor
ACL	anterior cruciate ligament
PET	polyethylene terephthalate
BMP-7	bone morphogenetic protein 7
LbL	layer-by-layer
PET	polyethylene terephthalate
PGS	poly (glycerol sebacate)
KGN	Kartogenin
MSC	mesenchymal stem/stromal cells
CNFs	carbon nanofibers
PAN	polyacrylonitrile
BMMSCs	bone marrow mesenchymal stem cells
GAC	glacial acetic acid
EC	ethylene carbonate
PTFE	polytetrafluoroethylene
(GO)NPs	graphene oxide nanoparticles
PCS	poly (citrates-siloxane)
PLGA	poly(lactic-co-glycolic acid)
HA	hydroxyapatite
Dex	examethasone
PNI	Peripheral nerve injuries
AFG/fSAP	aligned fibrin/functionalized self-assembling peptide
AChiG	Aligned chitosan nanofiber hydrogel
PLCL	poly(L-lactide-co-caprolactone)
TSF	tussah silk fibroin
ECs	endothelial cells
SMCs	smooth muscle cells
PLGATMC	poly(lactide–glycolide–trimethylene carbonate)
SDF-1α	stromal cell–derived factor-1-alpha
dPCU	degradable thermoplastic polycarbonate urethane
PTFE	polytetrafluoroethylene
GrGO	glucose (G)-reduced graphene oxide
GA	glutaraldehyde
AgNPs	silver nanoparticles
TCH	tetracycline hydrochloride
TC	tetracycline
PVA	poly(vinyl alcohol)
SS	silk sericin
AO	althea Officinalis
CA	calcium alginate
PDLCs	periodontal ligament cells
hPDLCs	human periodontal ligament cells
GT	gum tragacanth
SIS	small intestinal submucosa
ES	elastin
cin	cinnamon
CNC	cellulose nanocrystals
BCNC	bacterial cellulose nano-crystal
SSS	siliceous sponge spicules
PMS	poly (mannitol sebacate)
PDMS	polydimethylsiloxane
PVDFhfp	poly(vinylidene fluoride-co-hexafluoropropylene)
NT	N-terminal module
CT	C-terminal module
HUVEC	human umbilical vein endothelial cells
DOPA	dihydroxyphenylalanine
ADA	alginate dialdehyde
COL	collagen
HFBSCs	hair follicle bulge stem cells
PA	polyamide
NAC	N-Acetylcysteine
Eu	europium
